# Vibration Bandgap Tuning of Nonlinear Acoustic Metamaterials with Shape-Memory-Alloy Spring Oscillators Based on Effective-Negative-Mass Mechanism

**DOI:** 10.3390/ma19214446

**Published:** 2026-10-22

**Authors:** Ming Gao, Guodong Shang, Bin Fan, Linlin Sun, Dezhong Qi, Lingfeng Xu

**Affiliations:** 1 Mechanical and Electronic Engineering College, Shandong Agricultural University, Tai’an 271018, China; gaom@sdau.edu.cn (M.G.); 13615383504@163.com (G.S.); 2025120740@sdau.edu.cn (B.F.); sunlinlin@sdau.edu.cn (L.S.); 2 Shandong Engineering Research Center of Agricultural Equipment Intelligentization, Shandong Agricultural University, Tai’an 271018, China; 3 Shandong Higher Education Institution Future Industry Engineering Research Center of Intelligent Agricultural Robots, Shandong Agricultural University, Tai’an 271018, China; 4 Shandong Key Laboratory of Intelligent Production Technology and Equipment for Facility Horticulture, Shandong Agricultural University, Tai’an 271018, China

**Keywords:** acoustic metamaterial, bandgap tuning, effective negative mass, vibration attenuation, shape memory alloy

## Abstract

Traditional acoustic metamaterials with inherently fixed bandgaps face difficulty in satisfying the dynamic requirements of vibration isolation and vibration attenuation under varying operating conditions, which limits their practical engineering applications. Nonlinear acoustic metamaterials based on effective-negative-mass (ENM) mechanisms provide a promising approach for achieving adaptive vibration attenuation through bandgap regulation. In this study, a temperature-tunable nonlinear local-resonance acoustic metamaterial incorporating shape-memory-alloy (SMA) helical-spring oscillators is proposed. A positive-stiffness nonlinear acoustic metamaterial model is established by combining a nonlinear Duffing oscillator with SMA springs. Based on the ENM local-resonance theory, the governing equations are nondimensionalized, and the first-order perturbation solutions of the acoustic and optical dispersion branches are obtained using the perturbation method. The effects of temperature variation and stiffness ratio on the dispersion characteristics and the starting and cut-off frequencies of the bandgap are theoretically analyzed. The theoretical results show that, as the temperature decreases, the temperature-induced phase transformation of SMA reduces the stiffness of the SMA springs, causing both the starting and cut-off frequencies to decrease and the bandgap to shift toward lower frequencies and become narrower. Furthermore, a five-period finite structure model is established, and the numerical vibration-transmission results verify the theoretical predictions of temperature-dependent bandgap tuning. The dynamic responses further reveal that effective-positive-mass and ENM regions coexist within the bandgap, while a zero-effective-mass state occurs near the bandgap cut-off frequency. These findings demonstrate that the proposed SMA-based nonlinear acoustic metamaterial can actively regulate the low-frequency vibration attenuation range through temperature control, providing a theoretical basis for the design of adaptive acoustic metamaterials for vibration isolation and noise reduction.

## 1. Introduction

Acoustic metamaterials [1,2,3] are artificially engineered functional materials composed of periodic microstructured unit cells, which have attracted extensive attention for vibration attenuation, vibration isolation, and acoustic energy manipulation. Their unique bandgap characteristics enable the effective control of elastic wave propagation within specific frequency ranges [4]. Within the bandgap range, incident mechanical waves undergo strong reflection, attenuation, or even complete prohibition of propagation, thereby achieving effective vibration isolation and control for precision instruments and mechanical equipment [5,6]. The local-resonance mechanism [7,8] is widely used because it can efficiently manipulate low-frequency acoustic waves under subwavelength conditions, where the structural dimension is much smaller than the acoustic wavelength. This overcomes the limitation of conventional Bragg scattering, which requires the structural dimensions to be comparable to the wavelength. Therefore, the regulation of low-frequency attenuation bands is of considerable importance for extending acoustic metamaterials toward adaptive vibration and noise control applications. However, the bandgap characteristics of traditional acoustic metamaterials are entirely determined by their geometric parameters, material properties, and the inherent design of the unit cells. Once the structural parameters are defined, the starting frequency, cut-off frequency, and width of the bandgap remain permanently fixed. Such passive designs have limited adaptability under varying operating conditions, severely restricting their engineering practicality and level of intelligence in the field of precision vibration isolation. Therefore, achieving temperature-controlled active tuning of the bandgap has emerged as one of the most critical challenges urgently requiring a breakthrough in current acoustic metamaterial research for the vibration isolation of precision instruments and mechanical equipment.

To break through the limitations of traditional linear acoustic metamaterials, nonlinear acoustic metamaterials have emerged as an important research direction in recent years. Among these, a common approach is the introduction of a nonlinear restoring force into the local oscillator, such as a Duffing-type [9] cubic nonlinear term. This enables nonlinear acoustic metamaterials to exhibit ultra-low-frequency and ultra-broadband bandgap attenuation characteristics, significantly expanding the flexibility of low-frequency broadband vibration control for precision instruments and mechanical equipment. Sheng et al. [10] constructed a strongly nonlinear acoustic metamaterial beam and discovered that the chaotic band effect can substantially attenuate vibrations within the passband, while the added mass has a negligible impact on the vibration reduction performance. Based on this, they optimized a lightweight structure with an added mass ratio of only 28.1%, achieving low-frequency, broadband, and highly efficient vibration suppression. Taking an acoustic metamaterial consisting of an elastomeric matrix with circular holes as an example, Ning et al. [11] introduced large deformations via equibiaxial compression, revealing the distinct tuning effects of geometric and material nonlinearities on the local-resonance bandgap and the Bragg bandgap. Zhang et al. [12] designed a novel nonlinear acoustic metamaterial beam with two Duffing oscillators attached in series to its top. By analyzing the dispersion relation using the harmonic balance method, they found that introducing softening nonlinearity can achieve an ultra-broad bandgap starting from 0 Hz, providing a novel pathway for ultra-low-frequency broadband vibration suppression. Li et al. [13] used a double-layer thin-plate local-resonance acoustic metamaterial as a carrier to construct a multi-scale acoustic metamaterial for low-frequency broadband sound insulation. By replacing the homogeneous mass with a particle-filled mass in the local-resonance unit cells, they achieved excellent frequency and amplitude tuning capabilities in the low-frequency range. Zhang et al. [14] found that periodically mounting a structure composed of an inclined spring and two masses onto a beam increased the widths of the two local-resonance bandgaps of the nonlinear metamaterial beam, substantially broadening the bandgap range. Ou et al. [15] proposed a one-dimensional nonlinear acoustic metamaterial incorporating an inertial amplification mechanism. By introducing amplitude-dependent dynamic behaviors through geometric nonlinearity and spring cubic nonlinearity, and by using nonlinear modes to suppress linear resonance, they achieved ultra-low-frequency and ultra-broadband vibration reduction. The above studies demonstrate that the introduction of nonlinearity not only broadens the width of the bandgap but also provides new feasible methods for amplitude tuning and energy dissipation. Consequently, nonlinear acoustic metamaterials have become an important approach for low-frequency broadband vibration attenuation and noise control in precision instruments, mechanical equipment, and acoustic functional devices.

In nonlinear acoustic metamaterials, effective negative mass (ENM) [16,17] is one of the core physical mechanisms for achieving low-frequency bandgaps. It originates from the out-of-phase motion between the internal oscillators and the outer shells within the local-resonance unit cells. The resulting opposing inertial force counteracts the structural response induced by external excitation, thereby suppressing wave propagation and forming a bandgap. This mechanism allows acoustic metamaterials to block low-frequency elastic waves and reduce vibration transmission, which is highly desirable for vibration isolation and noise reduction applications. Hou et al. [18] established a temperature-dependent model of a one-dimensional spring–mass acoustic metamaterial and found that an increase in temperature causes the bandgap and the ENM region to shift toward lower frequencies while narrowing the bandwidth.

Furthermore, by using the temperature field, they successfully achieved the regulation of the vibration attenuation zones and the directional transmission control of elastic waves. Bhatt et al. [19] investigated acoustic metamaterials possessing simultaneous ENM and negative effective stiffness. They discovered that when the natural frequencies of the longitudinal and transverse resonators approach each other, the two attenuation peaks couple within the same bandgap to form dual attenuation peaks, thereby achieving a high level of spatial attenuation across the entire attenuation band. Wang et al. [20] designed a star-shaped single-phase acoustic metamaterial composed of spherical masses and arc-shaped ligaments. By using rotational, monopolar, and dipolar resonances, they simultaneously achieved negative effective-mass density, negative effective bulk modulus, and negative effective shear modulus. Additionally, leveraging the external-strain-induced reversible large deformation of the arc-shaped ligaments, they accomplished the preliminary tuning of the bandgap. Gao et al. [21] established a local-resonance metamaterial model containing pure cubic nonlinear oscillators. Through perturbation analysis, they discovered that softening and hardening nonlinearities shift the bandgap toward lower and higher frequencies, respectively. Furthermore, they pointed out the coexistence of ENM, effective positive mass, and effective zero-mass regions within the bandgap, noting that the effective positive mass can also achieve vibration attenuation, thereby broadening the understanding of bandgap mechanisms in nonlinear metamaterials. Gao et al. [22] established a bistable nonlinear acoustic metamaterial model incorporating negative-stiffness Duffing oscillators. Through perturbation analysis, they found that the bandgap width of the negative-stiffness system is broadened compared to that of its positive-stiffness counterpart. They also revealed the physical mechanism of the coexistence of effective positive, negative, and zero masses within the bandgap, demonstrating that the nonlinear negative-mass mechanism can achieve the dynamic evolution of the bandgap via amplitude tuning. This provides a theoretical foundation for the structural design of ultra-low-frequency vibration isolation in precision instruments and mechanical equipment.

Although nonlinear negative-mass acoustic metamaterials have significantly enhanced precision vibration isolation performance, their bandgap ranges remain passively fixed, making it difficult to meet the dynamic requirements of precision instruments and high-end mechanical equipment under varying operational conditions. However, as a typical smart material, shape memory alloys (SMAs) possess temperature-responsive phase-transformation characteristics, enabling continuous and reversible tuning of their elastic modulus and stiffness. Lv et al. [23] periodically distributed steel balls as lumped masses on a SMA beam. By using the temperature-controlled martensite–austenite phase transformation, they achieved a large-range tuning of the Bragg bandgap center frequency by approximately 73%, providing a theoretical basis for the broadband tunable vibration reduction design of SMA structures themselves. Yuan et al. [24] integrated SMA springs and electromagnets into a double-layer superstructure beam. By using temperature to control the stiffness and length of the SMA springs and electrical current to control the magnetic force of the electromagnets, they achieved switching between two operational modes: contact and separation. Liu et al. [25] integrated Ni-Ti SMA helical springs into a plate-type local-resonance metamaterial. By using the temperature-controlled martensite–austenite phase transformation to simultaneously alter the shear modulus and free height of the springs, they achieved continuous and reversible tuning of the flexural wave bandgap within the low-frequency range of 124 Hz to 226 Hz, and validated the effectiveness of this tuning mechanism through theoretical models, numerical simulations, and experiments. Li et al. [26] found that constructing a temperature-controlled quasi-zero-stiffness resonator using SMAs allows for substantial changes in the SMA elastic modulus by adjusting the temperature, thereby continuously tuning the low-frequency flexural wave bandgap of the metamaterial beam within the range of 26 Hz to 69 Hz. Jo et al. [27] proposed a temperature-controlled defect-mode phononic crystal geared toward tunable ultrasonic sensors. By integrating SMAs into the defect region of the phononic crystal and using temperature to drive the reversible phase transformation of the SMAs to alter their elastic modulus, they achieved real-time dynamic tuning of the defect band frequency. The results demonstrate that this structure can precisely adjust the defect band frequency within a specified frequency range, effectively enhancing the adaptability and operating bandwidth of ultrasonic sensors in dynamic environments. Mao et al. [28] constructed an anti-tetrachiral metamaterial using SMAs. By simultaneously altering the geometric configuration and material properties of the structure through the temperature-controlled phase transformation, they successfully shifted the bandgap center frequency downward, achieving reversible switching and wide-range tuning of the low-frequency broad bandgap. Candido de Sousa et al. [29] applied SMA springs to local-resonance metamaterials. By using temperature to regulate the SMA phase transformation and alter the elastic modulus, they achieved active tuning of the bandgap frequency. The introduction of SMAs into acoustic metamaterials provides a means for the temperature-driven active control of stiffness, thereby enhancing their adaptability in precision instruments and high-end mechanical equipment.

However, most existing studies are confined to linear acoustic metamaterials or nonlinear acoustic metamaterials with passively fixed bandgaps, which severely limits their adaptability in dynamic environments. Few studies have combined nonlinear Duffing oscillators with SMA springs. Sepehri et al. [30] investigated the manipulation of dispersion curves and stop-bands in weakly nonlinear monoatomic and diatomic phononic lattices using SMA helical springs, and further introduced auxiliary SMA ground springs to generate tunable low-frequency attenuation zones. However, their study mainly focused on the temperature-dependent wave propagation and stop-band characteristics of nonlinear lattice chains, without considering the local-resonance and ENM mechanisms. The temperature-dependent bandgap regulation of nonlinear local-resonance acoustic metamaterials from the perspective of effective positive, negative, and zero-mass characteristics, together with the corresponding finite-period vibration-transmission behavior, remains to be further investigated. To bridge this gap, this paper proposes a temperature-tunable nonlinear acoustic metamaterial by integrating SMA helical springs with Duffing oscillators based on the local-resonance-induced effective-negative-mass mechanism. This model exploits the reversible solid-state phase-transformation of SMAs: induced by changes in ambient temperature, the internal crystal structure of the SMA undergoes a reversible transformation between the low-stiffness martensite phase and the high-stiffness austenite phase, thereby causing a significant change in the equivalent stiffness of the spring. Leveraging this phase transformation characteristic, this paper achieves temperature-controlled tuning of the bandgap range of the acoustic metamaterial by varying the temperature, and the theoretical predictions are further validated through numerical simulations implemented in MATLAB R2012a. Furthermore, the temperature-dependent evolution of the effective positive, negative, and zero-mass states is systematically investigated, and their correspondence with the starting, local-resonance, and cut-off frequencies of the tunable bandgap is clarified through the dynamic responses of a five-period structure. This study provides a theoretical basis for the design of temperature-tunable nonlinear acoustic metamaterials.

The remainder of this paper is organized as follows: Section 2 introduces the temperature-driven phase-transformation behavior of SMAs. By introducing the martensite fraction and the rule of mixtures, a theoretical model for the equivalent elastic modulus of the material is constructed, and the temperature dependence of the SMA helical-spring stiffness is characterized. Section 3 considers a periodic structure incorporating SMA helical springs and conducts a force analysis on it to derive the dispersion curve equation. Using the perturbation method, the first-order perturbation solutions for the acoustic and optical branches are obtained, thereby determining the starting frequency and cut-off frequency of the bandgap. Additionally, the influence of the SMA helical spring’s temperature on the starting and cut-off frequencies of the bandgap is analyzed. Section 4 establishes a finite-period model to numerically verify the active tuning performance and complex dynamic responses. Finally, Section 5 summarizes the primary contributions and conclusions of this paper.

## 2. Temperature-Driven Phase-Transformation Behavior of SMAs

### 2.1. Temperature-Induced Phase Transformation and Elastic Modulus Variation in SMAs

SMAs are a class of smart materials capable of undergoing reversible crystal structure transformations under the application of temperature variations or external stress. Macroscopically, they exhibit unique shape memory effects and superelasticity. Microscopically, their core characteristic is the solid-state thermoelastic crystal structure transformation between austenite and martensite within a specific temperature range. Their phase-transformation behavior is typically defined by four characteristic temperatures: martensite finish temperature (*M_f_*), martensite start temperature (*M_s_*), austenite start temperature (*A_s_*), and austenite finish temperature (*A_f_*). To focus on the thermally driven modulation mechanisms and simplify the dynamic behavior, this study exclusively investigates low-stress conditions. Under such conditions, SMAs undergo solely temperature-induced phase transformations, neglecting the influence of stress-induced phase transformations.

The low-stress condition is adopted here as a modeling assumption to isolate the temperature-induced phase transformation of the SMA. The present reduced-order formulation does not resolve the detailed stress distribution within the SMA spring wire; therefore, possible stress-induced phase transformation is not considered. This assumption defines the applicable range of the present theoretical model.

The temperature-dependent SMA stiffness description adopted in this study is based on the thermal phase-transformation relations of the Brinson model [31]. This formulation is selected because it captures the phase-dependent elastic-property changes responsible for bandgap tuning, while avoiding the additional constitutive variables associated with stress-induced transformation and transformation-strain evolution. Its use is therefore consistent with the thermal tuning mechanism investigated here. A similar application of Brinson’s thermal-transformation relations has been reported in the theoretical and experimental study of temperature-tunable SMA-based metamaterials [32]. To characterize the extent of the phase transformation, the martensite fraction, denoted by ξ, is introduced, which takes values in the range of 0 to 1. The material transitions between a completely austenitic state (ξ=0) and a completely martensitic state (ξ=1). During the cooling process, namely the transformation from austenite to martensite, when the temperature *T* lies between *M_f_* and *M_s_*, the martensite fraction can be expressed as [30,32]:

(1) $$\xi =\frac{1-\xi_{0}}{2}\left[\cos\left(\pi \frac{T-M_{f}}{M_{s}-M_{f}}\right)+1\right]$$

Here, $\xi_{0}$ denotes the initial martensite fraction at the beginning of the cooling transformation. In the present study, the cooling process is assumed to start from a fully austenitic state above $M_{s}$; therefore, $\xi_{0}=0$. Under this initial condition, the general cooling relation reduces to the expression used in the subsequent analysis.

It should be noted that the cooling and heating transformations follow different temperature paths due to the thermal hysteresis of the SMA. Therefore, within the transformation-temperature range, the martensite fraction and the corresponding material modulus at a given temperature depend on the previous thermal history. In the subsequent dispersion and finite-period analyses, only the cooling branch is considered in order to focus on the fundamental temperature-dependent bandgap-tuning mechanism.

Conversely, during the heating process, namely the transformation from martensite to austenite, when the temperature *T* lies between *A_s_* and *A_f_*, the martensite fraction can be expressed as [30,32]:

(2) $$\xi =\frac{\xi_{0}}{2}\left[\cos\left(\pi \frac{T-A_{s}}{A_{f}-A_{s}}\right)+1\right]$$

Consequently, the equivalent elastic modulus of the SMA follows the rule of mixtures and is determined by weighting the volume fractions of the two phases:

(3) $$E\left(\xi \right)=E_{A}+\xi (E_{M}-E_{A})$$

In Equation (3), $E_{A}$ represents the elastic modulus of the fully austenitic phase, whereas $E_{M}$ denotes the elastic modulus of the fully martensitic phase. It is evident that adjusting the temperature can alter ξ, thereby enabling the continuous tuning of the SMA’s stiffness. This provides a fundamental basis for its application in tunable structures.

### 2.2. Temperature-Dependent Mechanical Behavior of SMA Helical Springs

In practical engineering applications, SMAs are commonly fabricated into helical springs to facilitate their integration into periodic structures. For the case of small deformations, the restoring force of the SMA spring can be linearly approximated as: *f_SMA_ = k_SMA_u*, where *u* represents the deformation and *k_SMA_* denotes the spring stiffness. This stiffness depends on the geometric parameters of the spring and the shear modulus of the material [33]:

(4) $$k_{SMA}(\xi )=\frac{d^{4}}{8D^{3}N}G(\xi )$$

This stiffness is driven by the phase-dependent shear modulus G(ξ), alongside the geometric properties of the spring: wire diameter *d*, coil diameter *D*, and active turns *N*. Assuming a constant Poisson’s ratio ν, the equivalent elastic modulus is derived from the shear modulus through the standard isotropic relation: $E\left(\xi \right)=2(1+\nu )G(\xi )$. At relatively low temperatures, the cooling process occurs, and the martensite fraction of the SMA spring is calculated according to Equation (1), as the temperature decreases from *M_s_* to *M_f_*. Conversely, at relatively high temperatures, the heating-induced phase transformation occurs, and the martensite fraction is obtained according to Equation (2). Although the qualitative wave propagation behavior of the SMA elements during both the cooling and heating states is fundamentally identical (both being based on the continuous stiffness-tuning mechanism), the temperature-dependent trends of their elastic moduli are precisely opposite due to the reversed directions of the phase transformations. To focus on the primary physical principles and render the subsequent analytical solutions more systematic, the following sections of this study will focus exclusively on the cooling process of the SMA elements (i.e., as the temperature gradually decreases from above *M_s_* to *M_f_*) to specifically investigate the acoustic dispersion relations and conduct dynamic validations. In the present theoretical model, temperature is treated as a prescribed and spatially uniform control parameter, and the corresponding SMA stiffness is evaluated under quasi-static thermal conditions. Therefore, the transient heat-transfer process, heating/cooling rate, and thermal time constant are not explicitly included. In a practical implementation, the SMA spring temperature may be adjusted by an appropriate thermal actuation method, such as resistive heating and controlled cooling. The achievable actuation bandwidth depends on the thermal properties, spring geometry, heat-transfer conditions, and temperature-control system. These transient thermal effects are beyond the scope of the present theoretical analysis and require further experimental investigation. It should be noted that finite temperature may also introduce stochastic thermal fluctuations in nonlinear lattice systems. For example, previous studies have incorporated finite-temperature effects through stochastic thermal fields and the corresponding noise correlation functions [34]. In contrast, the temperature considered in the present study serves as a prescribed external control parameter for the thermally induced martensite–austenite phase transformation of the SMA spring. Its effect is incorporated deterministically through the temperature-dependent material modulus and the resulting SMA spring stiffness. Therefore, the present work focuses on temperature-induced stiffness modulation and the corresponding regulation of local-resonance and bandgap characteristics, rather than on stochastic thermal fluctuations. The influence of thermal noise and its correlation characteristics on the wave propagation behavior of SMA-based nonlinear acoustic metamaterials remains an important topic for future investigation.

It should be noted that the present study adopts a reduced-order lumped-parameter model to focus on the fundamental temperature-dependent bandgap-tuning mechanism. The outer shell and internal resonator are represented by concentrated masses, while the connecting springs are modeled as equivalent stiffness elements. Accordingly, the distributed mass of the SMA springs is not explicitly considered. In addition, the temperature-induced variation in the SMA spring free length and preload, the possible intermediate R-phase transformation, and the temperature dependence of the non-SMA springs are neglected. These assumptions allow the influence of the temperature-dependent SMA stiffness on the local resonance and bandgap characteristics to be isolated. Such effects may influence the quantitative dynamic response of a practical fabricated structure and will require further experimentally calibrated modeling in future work.

## 3. Perturbation Analysis of Dispersion Characteristics

Consider the introduction of SMA helical springs into the acoustic metamaterial alongside the incorporation of Duffing oscillators. Specifically, the spring connecting the internal mass and the outer shell is a nonlinear spring, and its restoring force is a linear combination of the relative displacement and the cube of the relative displacement with respect to the shell. Concurrently, SMA springs are embedded within the internal structure of the acoustic metamaterial.

In this section, normalized parameters are adopted to investigate the general influence of system parameters on the dispersion characteristics and bandgap evolution. The purpose of this theoretical analysis is to reveal the underlying physical mechanisms and parameter-dependent trends, rather than to directly reproduce the finite-period numerical model presented in Section 4.

**Figure 1:**
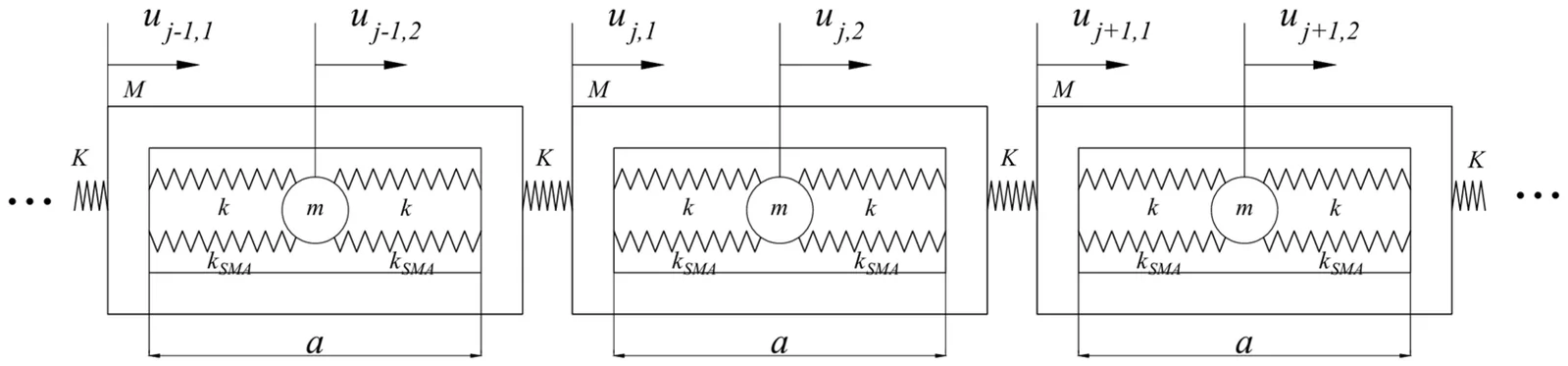
Periodic structure of ENM with SMA springs and Duffing oscillator.

Consider an infinite periodic structure as illustrated in Figure 1. The mass of the shell is *M*, and the stiffness coefficient of the spring connecting adjacent shells is *K*. The mass of the internal oscillator is *m*. This internal oscillator functions as a Duffing oscillator, where the linear stiffness coefficient of the spring connecting the Duffing oscillator and the shell is *k*, and the coefficient of the cubic displacement term is Γ. Simultaneously, a SMA spring with a stiffness of *k_SMA_* also connects the internal oscillator and the shell. For the *j*-th period cell, $u_{j,1}$ and $u_{j,2}$ denote the displacements of the shell and the Duffing oscillator of the *j*-th period cell, respectively. Furthermore, $u_{j-1,1}$ and $u_{j-1,2}$ represent the displacements of the shell and the internal Duffing oscillator of the (*j* − 1)-th period cell, respectively, while $u_{j+1,1}$ and $u_{j+1,2}$ represent the displacements of the shell and the internal Duffing oscillator of the (*j* + 1)-th period cell, respectively.

When *k* > 0, the sum of the internal nonlinear spring coefficient and the total stiffness coefficient of the SMA is greater than zero, i.e., (*k + k_SMA_*) > 0. For the shell of the *j*-th period cell, it is subjected not only to the force exerted by the internal Duffing oscillator, but also to the interaction forces from the shells of the (*j* + 1)-th and (*j* − 1)-th period cells. Simultaneously, it is acted upon by the restoring force from the internal SMA helical spring. Consequently, the differential equation of motion is formulated as follows:

In the present SMA–Duffing unit cell, the internal oscillator is coupled to the shell through the Duffing spring and the SMA helical spring in parallel. Accordingly, the linear restoring contribution is governed by the combined stiffness *k + k_SMA_*(*T*), while the cubic nonlinear contribution is determined by the Duffing term. For the shell of the *j*-th period cell, it is subjected not only to the interaction forces from the adjacent shells, but also to the restoring forces provided by the internal Duffing spring and the SMA helical spring. Consequently, the differential equation of motion is formulated as follows:

(5) $$M{\ddot{u}}_{j,1}+K(2u_{j,1}-u_{j-1,1}-u_{j+1,1})-2k(u_{j,2}-u_{j,1})-2\varepsilon \Gamma {\left(u_{j,2}-u_{j,1}\right)}^{3}-2k_{SMA}(u_{j,2}-u_{j,1})=0$$

Based on the force characteristics of the internal Duffing oscillator, it is solely subjected to the interaction force exerted by the shell of the *j*-th period cell and the restoring force provided by the SMA spring. Consequently, its equation of motion is expressed as follows:

(6) $$m{\ddot{u}}_{j,2}+2k(u_{j,2}-u_{j,1})+2\varepsilon \Gamma {\left(u_{j,2}-u_{j,1}\right)}^{3}+2k_{SMA}(u_{j,2}-u_{j,1})=0$$

It should be noted that the reduced governing equations in Equation (6) share a similar mathematical framework with the coupled nonlinear oscillator models reported previously [35,36]. Karabalin et al. [35] investigated the nonlinear dynamics of two elastically coupled nano-mechanical resonators, while Chotorlishvili et al. [36] further analyzed the nonlinear resonance characteristics and energy transfer between coupled oscillators with different coupling strengths. These studies mainly focused on amplitude–frequency responses, nonlinear resonance, and energy exchange behaviors of coupled oscillators.

In contrast, Equation (6) in the present work represents the dynamic equation of a unit cell in a periodic mass-in-mass acoustic metamaterial. The nonlinear interaction originates from the relative displacement between the internal resonator and the outer shell, and the outer shell is further coupled with neighboring cells through the inter-cell spring. Therefore, the present study focuses on nonlinear dispersion characteristics, locally resonant bandgap tuning, and effective-mass behavior rather than the nonlinear vibration response of two isolated coupled resonators. Although the mathematical framework is related to previous coupled oscillator models, the physical configuration and investigated phenomena are different.

It should be noted that the coupling strength between oscillators plays an important role in coupled nonlinear systems. In previous studies of coupled nano-mechanical resonators, the connectivity parameter was introduced to characterize the coupling intensity between two oscillators [35,36]. A larger connectivity indicates stronger interaction and energy exchange between the coupled oscillators.

In the present mass-in-mass acoustic metamaterial, the coupling mechanism is different from that of two isolated coupled resonators. The internal mass and the outer shell constitute a local resonator, while the shell is coupled with neighboring unit cells through the inter-cell stiffness *K*. Therefore, the coupling effect is reflected through the lattice coupling stiffness *K* and the local-resonance parameters rather than an independent connectivity parameter. In this work, we focus on the temperature-dependent modulation of the local-resonance frequency induced by the SMA spring, while the coupling topology of the periodic lattice remains unchanged.

Expressing the above Equations (5) and (6) in matrix form yields:

(7) $$\left[\begin{array}{cc} M & 0\\ 0 & m \end{array}\right]\left[\begin{array}{c} {\ddot{u}}_{j,1}\\ {\ddot{u}}_{j,2} \end{array}\right]+\left[\begin{array}{cc} 2(K+k+k_{SMA}) & -2(k+k_{SMA})\\ -2(k+k_{SMA}) & 2(k+k_{SMA}) \end{array}\right]\left[\begin{array}{c} u_{j,1}\\ u_{j,2} \end{array}\right]-\left[\begin{array}{c} K(u_{j-1,1}+u_{j+1,1})\\ 0 \end{array}\right]+2\varepsilon \Gamma \left[\begin{array}{c} -{\left(u_{j,2}-u_{j,1}\right)}^{3}\\ {\left(u_{j,2}-u_{j,1}\right)}^{3} \end{array}\right]=0$$

where ε is a small nonlinear perturbation parameter, ε≪1.

Defining the linear stiffness ratio between the shell and the nonlinear spring as $\alpha =\frac{K}{k}$, the stiffness ratio between the SMA helical spring and the nonlinear spring as $\eta =\frac{k_{SMA}}{k}$, the mass ratio as $\beta =\frac{M}{m}$, and the linear natural frequency as $\omega_{n}=\sqrt{\frac{2k}{m}}$, and by introducing the dimensionless time τ=ωt and the normalized cubic nonlinear coefficient $\bar{\Gamma }=\frac{\Gamma }{m\omega_{n}^{2}}$, Equation (7) can be nondimensionalized as follows:

(8) $${\bar{\omega }}^{2}\left[\begin{array}{cc} \beta & 0\\ 0 & 1 \end{array}\right]\left[\begin{array}{c} \frac{d^{2}u_{j,1}}{d\tau^{2}}\\ \frac{d^{2}u_{j,2}}{d\tau^{2}} \end{array}\right]+\left[\begin{array}{cc} 1+\alpha +\eta & -(1+\eta )\\ -(1+\eta ) & 1+\eta \end{array}\right]\left[\begin{array}{c} u_{j,1}\\ u_{j,2} \end{array}\right]-\frac{\alpha }{2}\left[\begin{array}{c} u_{j-1,1}+u_{j+1,1}\\ 0 \end{array}\right]+2\varepsilon \bar{\Gamma }\left[\begin{array}{c} -{\left(u_{j,2}-u_{j,1}\right)}^{3}\\ {\left(u_{j,2}-u_{j,1}\right)}^{3} \end{array}\right]=0$$

where $\bar{\omega }=\frac{\omega }{\omega_{n}}$ denotes the dimensionless frequency. Subsequently, the perturbation method is applied to derive the first-order perturbation solution for $\bar{\omega }$.

Using the following asymptotic expansion:

(9) $$\left\{\begin{array}{l} u_{j}=u_{j}^{\left(0\right)}+\varepsilon u_{j}^{\left(1\right)}+o(\varepsilon^{2})\\ \bar{\omega }={\bar{\omega }}_{0}+\varepsilon {\bar{\omega }}_{1}+o(\varepsilon^{2}) \end{array}\right.$$

Substituting Equation (9) into Equation (8):

(10) $$\begin{array}{c} {\left({\bar{\omega }}_{0}+\varepsilon {\bar{\omega }}_{1}\right)}^{2}\left[\begin{array}{cc} \beta & 0\\ 0 & 1 \end{array}\right]\left[\begin{array}{c} \frac{d^{2}u_{j,1}^{\left(0\right)}}{d\tau^{2}}+\varepsilon \frac{d^{2}u_{j,1}^{\left(1\right)}}{d\tau^{2}}\\ \frac{d^{2}u_{j,2}^{\left(0\right)}}{d\tau^{2}}+\varepsilon \frac{d^{2}u_{j,2}^{\left(1\right)}}{d\tau^{2}} \end{array}\right]+\left[\begin{array}{cc} 1+\alpha +\eta & -(1+\eta )\\ -(1+\eta ) & 1+\eta \end{array}\right]\left[\begin{array}{c} u_{j,1}^{\left(0\right)}+\varepsilon u_{j,1}^{\left(1\right)}\\ u_{j,2}^{\left(0\right)}+\varepsilon u_{j,2}^{\left(1\right)} \end{array}\right]\\ -\frac{\alpha }{2}\left[\begin{array}{c} u_{j-1,1}^{\left(0\right)}+u_{j+1,1}^{\left(0\right)}+\varepsilon (u_{j-1,1}^{\left(1\right)}+u_{j+1,1}^{\left(1\right)})\\ 0 \end{array}\right]+2\varepsilon \bar{\Gamma }\left[\begin{array}{c} -{\left(u_{j,2}^{\left(0\right)}-u_{j,1}^{\left(0\right)}\right)}^{3}\\ {\left(u_{j,2}^{\left(0\right)}-u_{j,1}^{\left(0\right)}\right)}^{3} \end{array}\right]+o(\varepsilon^{2})=0 \end{array}$$

Separating the coefficients of $\varepsilon^{0}$ and $\varepsilon^{1}$ and equating them to zero yields:

$\varepsilon^{0}$ coefficient:

(11) $${\bar{\omega }}_{0}^{2}\left[\begin{array}{cc} \beta & 0\\ 0 & 1 \end{array}\right]\left[\begin{array}{c} \frac{d^{2}u_{j,1}^{\left(0\right)}}{d\tau^{2}}\\ \frac{d^{2}u_{j,2}^{\left(0\right)}}{d\tau^{2}} \end{array}\right]+\left[\begin{array}{cc} 1+\alpha +\eta & -(1+\eta )\\ -(1+\eta ) & 1+\eta \end{array}\right]\left[\begin{array}{c} u_{j,1}^{\left(0\right)}\\ u_{j,2}^{\left(0\right)} \end{array}\right]-\frac{\alpha }{2}\left[\begin{array}{c} u_{j-1,1}^{\left(0\right)}+u_{j+1,1}^{\left(0\right)}\\ 0 \end{array}\right]=0$$

$\varepsilon^{1}$ coefficient:

(12) $$\begin{array}{c} {\bar{\omega }}_{0}^{2}\left[\begin{array}{cc} \beta & 0\\ 0 & 1 \end{array}\right]\left[\begin{array}{c} \frac{d^{2}u_{j,1}^{\left(1\right)}}{d\tau^{2}}\\ \frac{d^{2}u_{j,2}^{\left(1\right)}}{d\tau^{2}} \end{array}\right]+\left[\begin{array}{cc} 1+\alpha +\eta & -(1+\eta )\\ -(1+\eta ) & 1+\eta \end{array}\right]\left[\begin{array}{c} u_{j,1}^{\left(1\right)}\\ u_{j,2}^{\left(1\right)} \end{array}\right]-\frac{\alpha }{2}\left[\begin{array}{c} u_{j-1,1}^{\left(1\right)}+u_{j+1,1}^{\left(1\right)}\\ 0 \end{array}\right]\\ +2{\bar{\omega }}_{0}{\bar{\omega }}_{1}\left[\begin{array}{cc} \beta & 0\\ 0 & 1 \end{array}\right]\left[\begin{array}{c} \frac{d^{2}u_{j,1}^{\left(0\right)}}{d\tau^{2}}\\ \frac{d^{2}u_{j,2}^{\left(0\right)}}{d\tau^{2}} \end{array}\right]+2\bar{\Gamma }\left[\begin{array}{c} -{\left(u_{j,2}^{\left(0\right)}-u_{j,1}^{\left(0\right)}\right)}^{3}\\ {\left(u_{j,2}^{\left(0\right)}-u_{j,1}^{\left(0\right)}\right)}^{3} \end{array}\right]=0 \end{array}$$

### 3.1. Linear Dispersion Curve Equation

The steady-state solution of Equation (11) assumes the following form:

(13) $$\left[\begin{array}{c} \begin{array}{l} u_{j,1}^{\left(0\right)}\\ u_{j,2}^{\left(0\right)} \end{array} \end{array}\right]=\left[\begin{array}{c} A_{1}^{\left(0\right)}\\ A_{2}^{\left(0\right)} \end{array}\right]\frac{1}{2}e^{iqaj}e^{i\tau }+c.c.$$

(14) $$\left[\begin{array}{c} \begin{array}{l} u_{j\pm 1,1}^{\left(0\right)}\\ u_{j\pm 1,2}^{\left(0\right)} \end{array} \end{array}\right]=\left[\begin{array}{c} A_{1}^{\left(0\right)}\\ A_{2}^{\left(0\right)} \end{array}\right]\frac{1}{2}e^{iqa(j\pm 1)}e^{i\tau }+c.c.$$

where *a* denotes the distance between two adjacent shells, i.e., the lattice constant of the periodic structure; *q* is the Bloch wavenumber, and j denotes the index of the period cell. Therefore, *qaj* represents the spatial phase associated with the *j*-th period cell, while *e^iqaj^* denotes the corresponding spatial phase factor. $A_{1}^{\left(0\right)}$ and $A_{2}^{\left(0\right)}$ represent the zeroth-order steady-state complex amplitudes of the shell and the internal oscillator in the *j*-th period cell, respectively, and c.c. denotes the complex conjugate of the preceding positive-exponential term. Substituting Equations (13) and (14) into Equation (11):

(15) $$\left[\begin{array}{cc} -{\bar{\omega }}_{0}^{2}\beta +(1+\eta )+2\alpha \sin^{2}(\frac{qa}{2}) & -(1+\eta )\\ -(1+\eta ) & (1+\eta )-{\bar{\omega }}_{0}^{2} \end{array}\right]\left[\begin{array}{c} A_{1}^{\left(0\right)}\\ A_{2}^{\left(0\right)} \end{array}\right]\frac{1}{2}e^{iqaj}e^{i\tau }+c.c.=0$$

For Equation (15) to yield non-trivial solutions for $A_{1}^{\left(0\right)}$ and $A_{2}^{\left(0\right)}$, the determinant of its coefficient matrix must be equal to zero; hence,

(16) $$\left(\beta +\frac{1+\eta }{(1+\eta )-{\bar{\omega }}_{0}^{2}}){\bar{\omega }}_{0}^{2}=2\alpha \sin^{2}(\frac{qa}{2}\right)$$

$A_{1}^{\left(0\right)}$ and $A_{2}^{\left(0\right)}$ satisfy the following relation:

(17) $$A_{1}^{\left(0\right)}=\frac{(1+\eta )-{\bar{\omega }}_{0}^{2}}{1+\eta }A_{2}^{\left(0\right)}$$

Solving Equation (16) for $A_{1}^{\left(0\right)}$ and ${\bar{\omega }}_{0}$ yields the dispersion equations for the two frequency branches:

(18) $${\bar{\omega }}_{0}^{aco}=\sqrt{\frac{\left[\left(\beta +1)(1+\eta )+2\alpha \sin^{2}(\frac{qa}{2}\right)\right]-\sqrt{{\left[\left(\beta +1)(1+\eta )+2\alpha \sin^{2}(\frac{qa}{2}\right)\right]}^{2}-8\alpha \beta (1+\eta )\sin^{2}(\frac{qa}{2})}}{2\beta }}$$

(19) $${\bar{\omega }}_{0}^{opt}=\sqrt{\frac{\left[\left(\beta +1)(1+\eta )+2\alpha \sin^{2}(\frac{qa}{2}\right)\right]+\sqrt{{\left[\left(\beta +1)(1+\eta )+2\alpha \sin^{2}(\frac{qa}{2}\right)\right]}^{2}-8\alpha \beta (1+\eta )\sin^{2}(\frac{qa}{2})}}{2\beta }}$$

where ${\bar{\omega }}_{0}^{aco}$ and ${\bar{\omega }}_{0}^{opt}$ denote the acoustic and optical branches, respectively. The acoustic branch corresponds to the lower-frequency dispersion curve, whereas the optical branch corresponds to the higher-frequency dispersion curve.

Equations (18) and (19) show that, in the linear limit, the SMA spring is mechanically in parallel with the linear component of the Duffing spring. Its contribution therefore enters the governing equations only through the total linear stiffness *k*(*T*) = *k* + *k_SMA_*(*T*), which is represented by (1 + η) in the nondimensional formulation. Accordingly, the linear dispersion problem considered here is equivalent to that in Refs. under the replacement *k* + *k_SMA_*(*T*). Therefore, the temperature-induced phase transformation of the SMA changes *k_SMA_*(*T*), leading to corresponding shifts in both the acoustic and optical branches.

### 3.2. Nonlinear Dispersion Curve Equation

Next, we consider the first-order perturbation solution. For Equation (12), the solution takes the form:

(20) $$\left[\begin{array}{c} u_{j,1}^{\left(1\right)}\\ u_{j,2}^{\left(1\right)} \end{array}\right]=\left[\begin{array}{c} A_{1}^{\left(1\right)}\\ A_{2}^{\left(1\right)} \end{array}\right]\frac{1}{2}e^{iqaj}e^{i\tau }+c.c.$$

(21) $$\left[\begin{array}{c} u_{j\pm 1,1}^{\left(1\right)}\\ u_{j\pm 1,2}^{\left(1\right)} \end{array}\right]=\left[\begin{array}{c} A_{1}^{\left(1\right)}\\ A_{2}^{\left(1\right)} \end{array}\right]\frac{1}{2}e^{iqa(j\pm 1)}e^{i\tau }+c.c.$$

Substituting Equations (20) and (21) into Equation (12) yields:

(22) $$\begin{array}{l} \left[\begin{array}{cc} -{\bar{\omega }}_{0}^{2}\beta +1+\eta +2\alpha \sin^{2}(\frac{qa}{2}) & -(1+\eta )\\ -(1+\eta ) & 1+\eta -{\bar{\omega }}_{0}^{2} \end{array}\right]\left[\begin{array}{c} A_{1}^{\left(1\right)}\\ A_{2}^{\left(1\right)} \end{array}\right]\frac{1}{2}e^{iqaj}e^{i\tau }+c.c.=\left[\begin{array}{c} {\bar{\omega }}_{0}{\bar{\omega }}_{1}\beta A_{1}^{\left(0\right)}+2\bar{\Gamma }N^{\left(0\right)}\\ {\bar{\omega }}_{0}{\bar{\omega }}_{1}A_{2}^{\left(0\right)}-2\bar{\Gamma }N^{\left(0\right)} \end{array}\right]\frac{1}{2}e^{iqaj}e^{i\tau }\\ +\left[\begin{array}{c} A_{1}^{\left(0\right)}\\ A_{2}^{\left(0\right)} \end{array}\right]Ce^{3iqaj}e^{3i\tau }+c.c. \end{array}$$

The cubic term in Equation (7) originates from the relative displacement ${\left(u_{j,2}-u_{j,1}\right)}^{3}$ and represents an internal action–reaction pair between the shell and the internal oscillator. It therefore has opposite signs in the two equations. After substituting the first-order expansions, ${\left(u_{j,2}-u_{j,1}\right)}^{3}$ produces a fundamental-frequency (secular) component and a third-harmonic (non-secular) component. Only the fundamental-frequency component is retained in the secular-term elimination for the first-order correction; the third-harmonic component does not participate in this order. The sign convention in Equation (22) follows directly from Equation (7).

Where $N^{\left(0\right)}=\frac{3}{4}({\bar{A}}_{2}^{\left(0\right)}-{\bar{A}}_{1}^{\left(0\right)}){\left(A_{2}^{\left(0\right)}-A_{1}^{\left(0\right)}\right)}^{2}$.

To eliminate the secular terms, equating only the coefficients of *e^iqaj^* and $e^{i\tau }$ on both sides of Equation (22) yields:

(23) $$\left[\begin{array}{cc} -{\bar{\omega }}_{0}^{2}\beta +1+\eta +2\alpha \sin^{2}(\frac{qa}{2}) & -(1+\eta )\\ -(1+\eta ) & 1+\eta -{\bar{\omega }}_{0}^{2} \end{array}\right]\left[\begin{array}{c} A_{1}^{\left(1\right)}\\ A_{2}^{\left(1\right)} \end{array}\right]=\left[\begin{array}{c} {\bar{\omega }}_{0}{\bar{\omega }}_{1}\beta A_{1}^{\left(0\right)}+2\bar{\Gamma }N^{\left(0\right)}\\ {\bar{\omega }}_{0}{\bar{\omega }}_{1}A_{2}^{\left(0\right)}-2\bar{\Gamma }N^{\left(0\right)} \end{array}\right]$$

Letting $C_{1}=-\beta {\bar{\omega }}_{0}^{2}+1+\eta +2\alpha \sin^{2}(\frac{qa}{2})$ and considering Equation (16), we obtain:

(24) $$C_{1}=\frac{{\left(1+\eta \right)}^{2}}{(1+\eta )-{\bar{\omega }}_{0}^{2}}$$

Applying linear transformations to the augmented matrix of Equation (23) yields:

(25) $$\left[\begin{array}{lll} C_{1} & -(1+\eta ) & {\bar{\omega }}_{0}{\bar{\omega }}_{1}\beta A_{1}^{\left(0\right)}+2\bar{\Gamma }N^{\left(0\right)}\\ 0 & 0 & \left({\bar{\omega }}_{0}{\bar{\omega }}_{1}A_{2}^{\left(0\right)}-2\bar{\Gamma }N^{\left(0\right)}\right)+\frac{\left(1+\eta \right)-{\bar{\omega }}_{0}^{2}}{1+\eta }\left({\bar{\omega }}_{0}{\bar{\omega }}_{1}\beta A_{1}^{\left(0\right)}+2\bar{\Gamma }N^{\left(0\right)}\right) \end{array}\right]$$

For Equation (25) to yield non-trivial solutions for $A_{1}^{\left(1\right)}$ and $A_{2}^{\left(1\right)}$, the rank of its coefficient matrix must be equal to that of its augmented matrix; hence,

(26) $$({\bar{\omega }}_{0}{\bar{\omega }}_{1}A_{2}^{\left(0\right)}-2\bar{\Gamma }N^{\left(0\right)})+\frac{(1+\eta )-{\bar{\omega }}_{0}^{2}}{1+\eta }({\bar{\omega }}_{0}{\bar{\omega }}_{1}\beta A_{1}^{\left(0\right)}+2\bar{\Gamma }N^{\left(0\right)})=0$$

The solution is:

(27) $${\bar{\omega }}_{1}=\frac{2\bar{\Gamma }N^{\left(0\right)}{\bar{\omega }}_{0}}{(1+\eta )A_{2}^{\left(0\right)}+A_{1}^{\left(0\right)}\beta \left[(1+\eta )-{\bar{\omega }}_{0}^{2}\right]}$$

Therefore, the perturbation solution takes the form:

(28) $$\bar{\omega }={\bar{\omega }}_{0}+\varepsilon {\bar{\omega }}_{1}={\bar{\omega }}_{0}+\varepsilon \frac{2\bar{\Gamma }N^{\left(0\right)}{\bar{\omega }}_{0}}{(1+\eta )A_{2}^{\left(0\right)}+A_{1}^{\left(0\right)}\beta \left[(1+\eta )-{\bar{\omega }}_{0}^{2}\right]}+O(\varepsilon^{2})$$

Given the relationship between $A_{1}^{\left(0\right)}$ and $A_{2}^{\left(0\right)}$, Equation (28) can be written as:

(29) $$\bar{\omega }={\bar{\omega }}_{0}+\varepsilon \frac{3\bar{\Gamma }{\left|A_{2}^{\left(0\right)}\right|}^{2}{\bar{\omega }}_{0}^{7}}{2{\left(1+\eta \right)}^{2}\left\{{\left(1+\eta \right)}^{2}+\beta {\left[(1+\eta )-{\bar{\omega }}_{0}^{2}\right]}^{2}\right\}}+O(\varepsilon^{2})$$

Substituting ${\bar{\omega }}_{0}^{opt}$ and ${\bar{\omega }}_{0}^{aco}$ into the above equation yields the corresponding first-order asymptotic expansion equations for the acoustic and optical dispersion curves:

(30) $${\bar{\omega }}_{opt}={\bar{\omega }}_{0}^{opt}+\varepsilon \frac{3\bar{\Gamma }{\left|A_{2}^{\left(0\right)}\right|}^{2}{\left({\bar{\omega }}_{0}^{opt}\right)}^{7}}{2{\left(1+\eta \right)}^{2}\left\{{\left(1+\eta \right)}^{2}+\beta {\left[(1+\eta )-{\left({\bar{\omega }}_{0}^{opt}\right)}^{2}\right]}^{2}\right\}}$$

(31) $${\bar{\omega }}_{aco}={\bar{\omega }}_{0}^{aco}+\varepsilon \frac{3\bar{\Gamma }{\left|A_{2}^{\left(0\right)}\right|}^{2}{\left({\bar{\omega }}_{0}^{aco}\right)}^{7}}{2{\left(1+\eta \right)}^{2}\left\{{\left(1+\eta \right)}^{2}+\beta {\left[(1+\eta )-{\left({\bar{\omega }}_{0}^{aco}\right)}^{2}\right]}^{2}\right\}}$$

Equations (30) and (31) represent the derived dispersion relations for the acoustic and optical branches, respectively. It can be seen from these dispersion relations that the dispersion characteristics are related to the stiffness ratio η between the SMA spring and the nonlinear spring. On the one hand, the SMA spring enables active shifting of the dispersion curves through its temperature-dependent stiffness; on the other hand, the Duffing nonlinearity further introduces amplitude dependence into this tuning process. The combined effects of these two mechanisms provide a theoretical basis for the subsequent multi-parameter active tuning of the bandgap position and width.

It should be noted that nonlinear acoustic metamaterials may exhibit complex dynamic behaviors, including nonlinear resonance interaction, bifurcation, and chaos, under strong excitation conditions [37]. However, the present study focuses on the weakly nonlinear regime, where the perturbation approach remains valid and the nonlinear term mainly contributes to the amplitude-dependent modification of dispersion characteristics. The chaotic transition boundaries associated with strong excitation, damping, and nonlinear parameters require further investigation.

**Figure 2:**
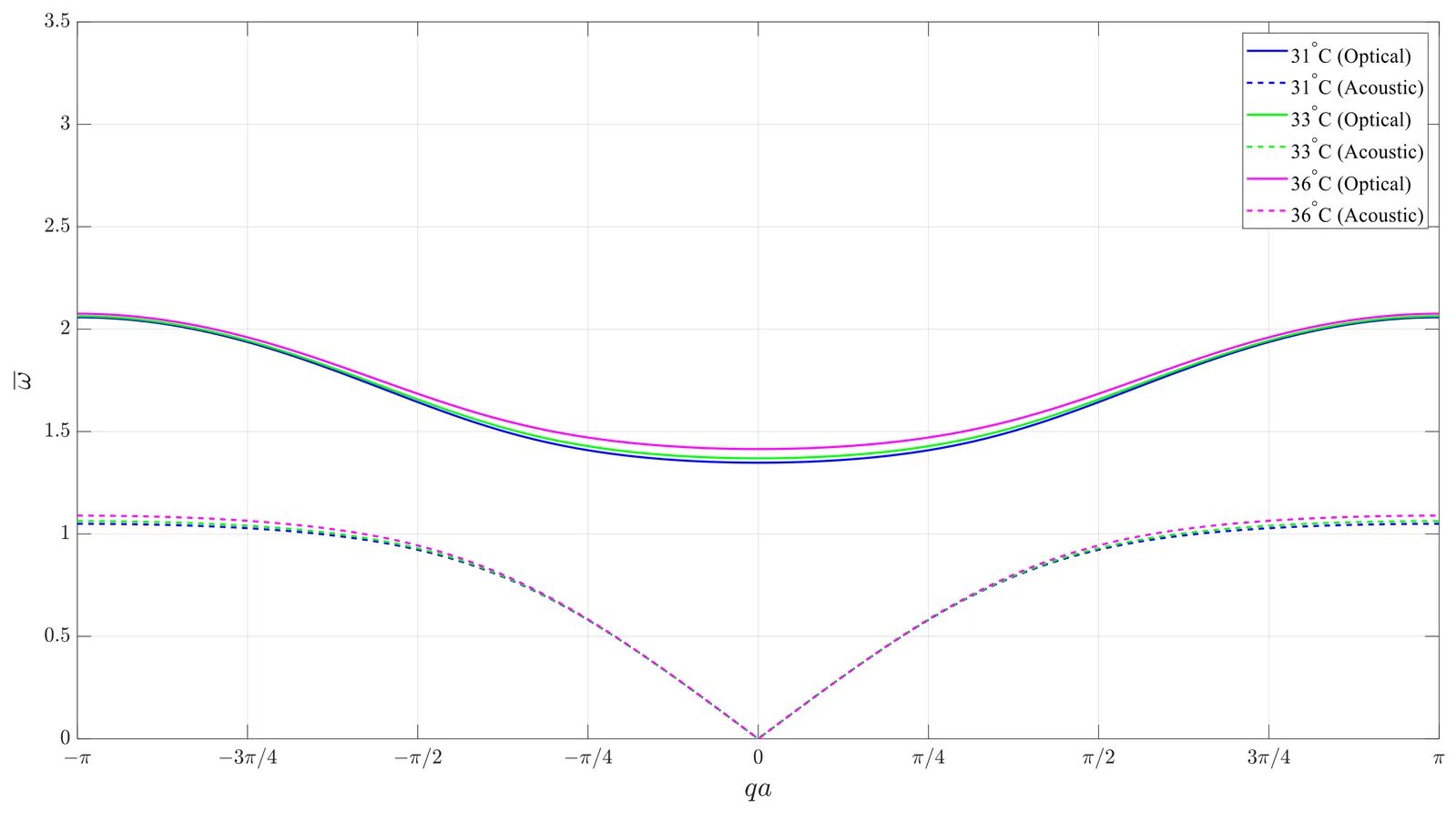
Acoustic dispersion curves at different temperatures.

Here, setting *α* = 5, *β* = 3, and *ε* = 0.0135, the magnitude of the zeroth-order amplitude of the internal oscillator is prescribed as $\left|A_{2}^{\left(0\right)}\right|=1$, and the coefficient in front of the nonlinear term is $\bar{\Gamma }=1$, and *η* is set to 0.28, 0.33, and 0.42. The characteristic temperatures are $M_{f}=29\;{}^{\circ}C$, $M_{s}=42\;{}^{\circ}C$, $A_{s}=43\;{}^{\circ}C$, and $A_{f}=58\;{}^{\circ}C$. The elastic moduli are *E_M_* = 35GPa and *E_A_* = 70GPa, and the Poisson’s ratio is ν=0.33 [29,30,38]. The SMA helical spring has a wire diameter of *d* = 0.001 m, a coil diameter of *D* = 0.01 m, and *N* = 50 active coils. The acoustic dispersion curves are plotted, as shown in Figure 2:

As can be seen from Figure 2, with the other structural parameters kept constant, both the acoustic and optical branches shift slightly toward the lower-frequency range as the temperature decreases. Although the dispersion curves at different temperatures remain close to one another, a consistent temperature-dependent shift can still be observed. This trend indicates that the temperature-dependent stiffness of the SMA spring has a relatively small but systematic influence on the dispersion characteristics of the periodic structure.

**Figure 3:**
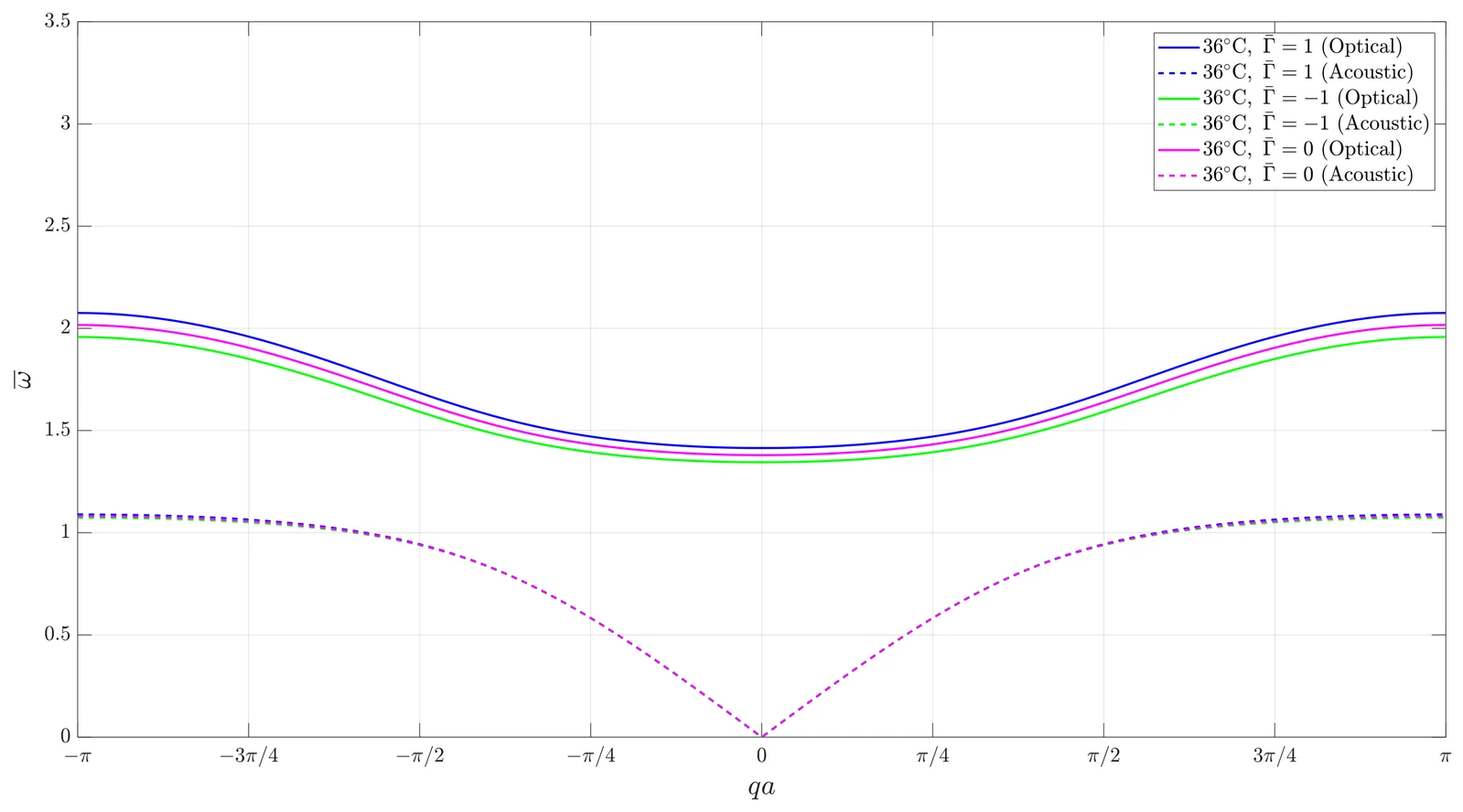
Effect of cubic nonlinearity on dispersion curves.

To further investigate the influence of the cubic nonlinear coefficient on the dispersion characteristics, the temperature is fixed at 36 °C, and the other structural and material parameters are kept consistent with those used in Figure 2. The magnitude of the zeroth-order amplitude of the internal oscillator is prescribed as $\left|A_{2}^{\left(0\right)}\right|=1$, and the perturbation parameter is set to *ε* = 0.0135. The dimensionless cubic nonlinear coefficient retains the sign of the original dimensional cubic stiffness coefficient because the reference scales used in the nondimensionalization are positive. Therefore, its positive and negative values correspond to hardening and softening nonlinearity, respectively, while a zero value corresponds to the linear case. Figure 3 presents the positive-stiffness acoustic and optical dispersion curves for $\bar{\Gamma }=1$, −1, and 0. The fixed temperature keeps the stiffness of the SMA spring unchanged during this comparison, allowing the influence of the cubic nonlinear coefficient to be examined separately. Here, 36 °C is selected as a representative temperature; the same analysis can be performed at other prescribed temperatures by recalculating the temperature-dependent stiffness of the SMA spring.

As shown in Figure 3, the cubic nonlinear coefficient modifies both the acoustic and optical dispersion branches. Compared with the linear reference curves obtained for $\bar{\Gamma }=0$, the positive coefficient $\bar{\Gamma }=1$ produces upward frequency corrections, indicating hardening nonlinearity, whereas the negative coefficient $\bar{\Gamma }=-1$ produces downward frequency corrections, indicating softening nonlinearity. Although the frequency shifts are relatively small within the present weakly nonlinear approximation, the comparison shows that the cubic nonlinear term controls the direction of the dispersion correction. Its contribution depends on both the cubic nonlinear coefficient and the squared amplitude of the internal oscillator, as indicated by the first-order dispersion expressions. Thus, Figure 3 illustrates the nonlinear contribution to the dispersion characteristics at a prescribed amplitude and fixed temperature.

### 3.3. Influence of Stiffness Ratio on Bandgap Range

For a linear acoustic dispersion curve ${\bar{\omega }}_{0}^{aco}$, when *qa* = π, the starting frequency of the bandgap is:

(32) $${\bar{\omega }}_{01}=\sqrt{\frac{\left[(\beta +1)(1+\eta )+2\alpha \right]-\sqrt{{\left[(\beta +1)(1+\eta )+2\alpha \right]}^{2}-8\alpha \beta (1+\eta )}}{2\beta }}$$

**Figure 4:**
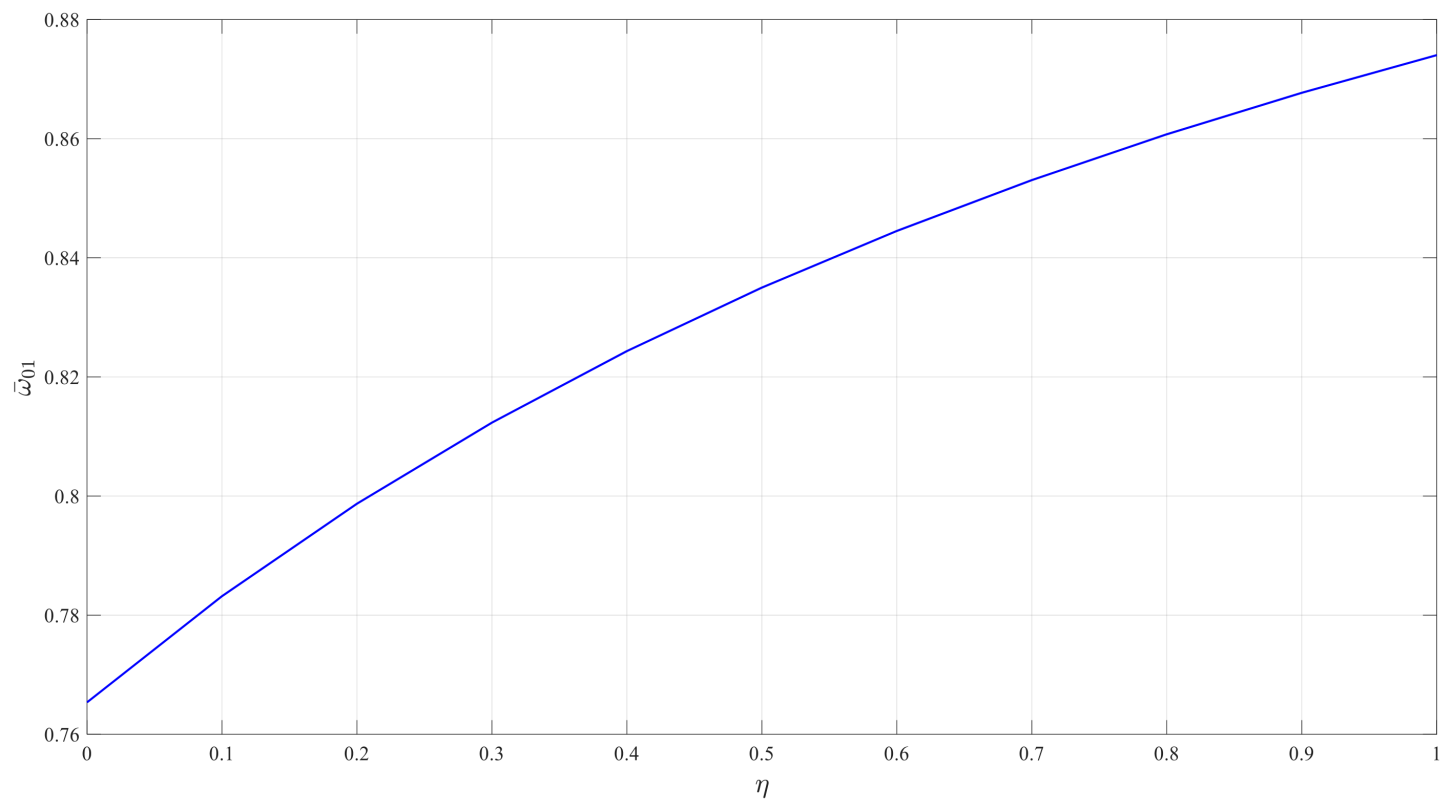
Effect of changes in the stiffness ratio η on the starting frequency ${\bar{\omega }}_{01}$.

Here, the parameters *α* = 1 and *β* = 1 are fixed to investigate the effect of variations in the stiffness ratio η on the starting frequency. The corresponding variation in the starting frequency is shown in Figure 4:

As can be observed from Figure 4, the dimensionless starting frequency ${\bar{\omega }}_{01}$ depends on the stiffness ratio between the SMA spring and the nonlinear spring. Furthermore, as η increases, the starting frequency rises accordingly.

For dispersion curve ${\bar{\omega }}_{0}^{opt}$ of the optical branch, when *qa* = 0, the cut-off frequency of the bandgap is:

(33) $$\begin{array}{l} {\bar{\omega }}_{02}=\sqrt{\frac{\left[\left(\beta +1)(1+\eta \right)\right]+\sqrt{{\left[\left(\beta +1)(1+\eta \right)\right]}^{2}}}{2\beta }}\\ =\sqrt{\frac{\left(\beta +1)(1+\eta \right)}{\beta }} \end{array}$$

**Figure 5:**
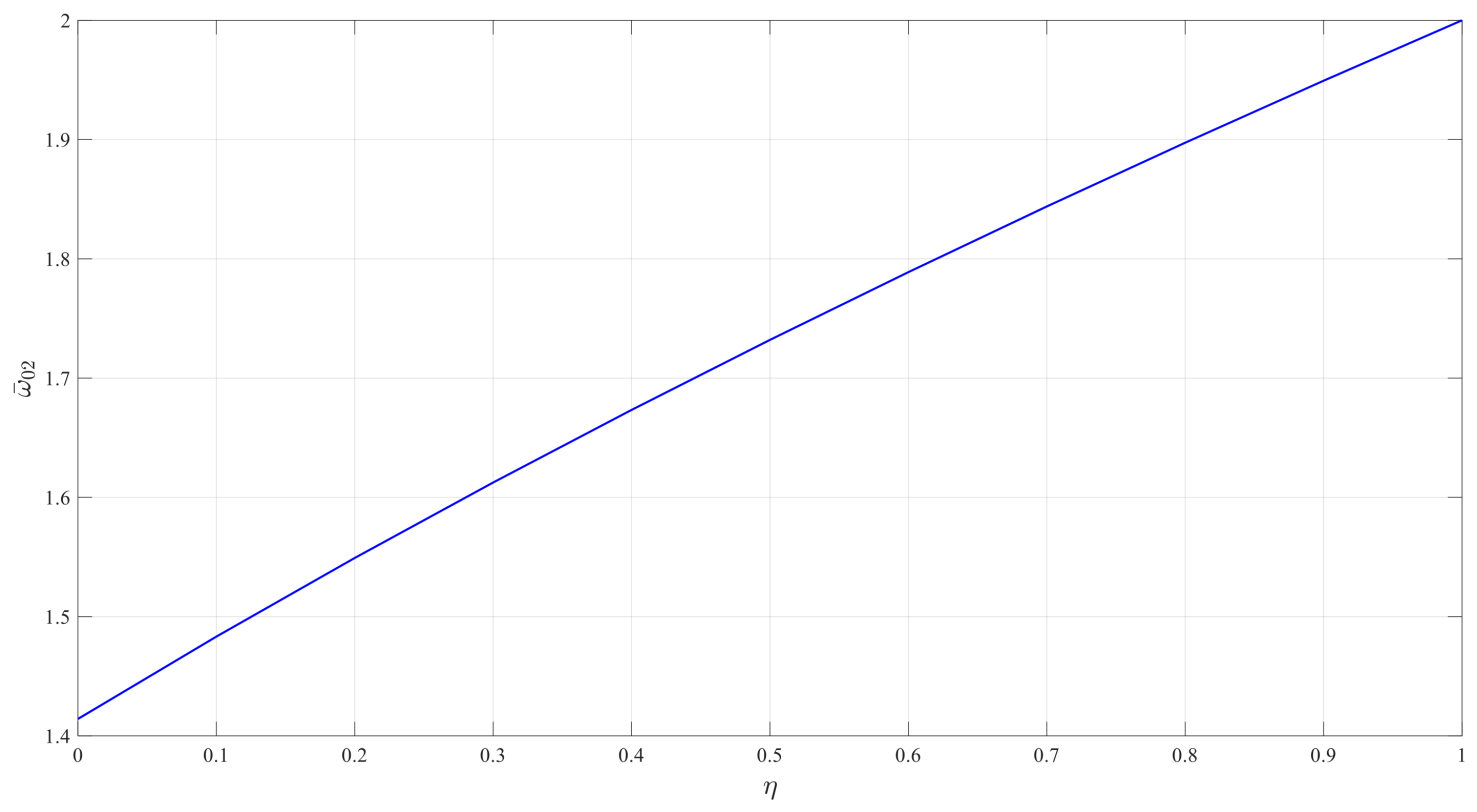
Effect of the variation in the stiffness ratio *η* on the cut-off frequency ${\bar{\omega }}_{01}$.

Here, the parameters *α* = 1 and *β* = 1 are fixed to investigate the effect of variations in the stiffness ratio *η* on the cut-off frequency. The corresponding variation curve is shown in Figure 5:

As can be seen from Equation (33), the cut-off frequency of the bandgap depends not only on the mass ratio *β* between the shell and the internal oscillator, but also on the stiffness ratio *η* between the SMA spring and the nonlinear spring. It can be further observed from Figure 5 that, as the stiffness ratio *η* increases, the cut-off frequency exhibits a continuous increasing trend with a relatively smooth variation. This indicates that the cut-off frequency can be continuously tuned by varying the stiffness of the SMA spring.

### 3.4. Effect of Temperature on Bandgap Characteristics

During the cooling process considered in this study, the martensite fraction of the SMA increases as the temperature decreases, resulting in reductions in its elastic modulus, shear modulus and, consequently, the stiffness of the SMA helical spring. Therefore, the stiffness ratio between the SMA spring and the nonlinear spring decreases with decreasing temperature.

According to the theoretical analysis in Section 3.3, both the starting and cut-off frequencies of the bandgap increase with the stiffness ratio. Hence, a reduction in temperature decreases both bandgap edge frequencies and shifts the bandgap toward the lower-frequency range. Moreover, the optical branch is more sensitive to the variation in SMA spring stiffness than the acoustic branch. Consequently, the cut-off frequency decreases more markedly than the starting frequency during cooling, leading to a reduction in the bandgap width. These results theoretically demonstrate that temperature variation can regulate the position and width of the bandgap by changing the stiffness of the SMA spring. The predicted temperature-dependent bandgap characteristics are further verified using the finite-period model in Section 4.

## 4. Numerical Verification of Bandgap-Tuning Characteristics

**Figure 6:**
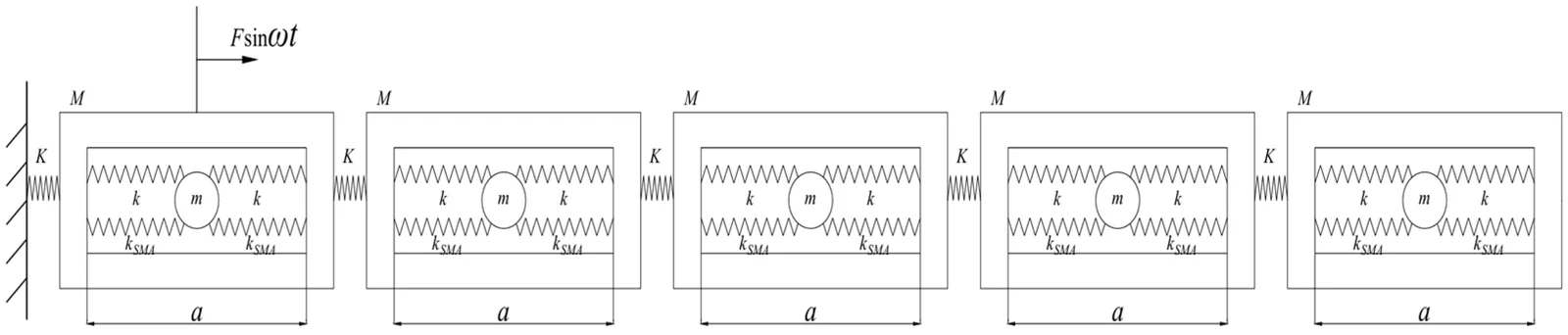
Five-period ENM system model incorporating SMA springs.

The preceding sections analyzed the bandgap characteristics of the infinite periodic acoustic metamaterial incorporating SMA springs. To verify the validity of the aforementioned theoretical analysis, a finite-period model of this acoustic metamaterial can be established. Therefore, a five-unit-cell ENM system model incorporating SMA springs is now established, as illustrated in Figure 6. A harmonic excitation force of Fsin(ωt) is applied to the shell of the first period cell, and the response is extracted at the shell of the fifth period cell. For each excitation frequency, the steady-state displacements of the shell and the Duffing oscillator in each period cell are calculated to obtain the vibration-transmission characteristics of this finite-period structure, thereby facilitating the analysis of the bandgap ranges and vibration-transmission behavior with respect to the temperature of the SMA springs.

The theoretical expressions of the bandgap starting and cut-off frequencies derived in Section 3 are further applied using the physical parameters of the finite-period structure. The comparison between analytical predictions and numerical transmission results demonstrates the validity of the proposed temperature-tuning mechanism. Due to the weakly nonlinear approximation adopted in the analytical derivation, slight differences between theoretical and numerical results are expected.

By analyzing the forces exerted on the shell and the internal oscillator of each period cell respectively, the system of differential equations of motion is established according to Newton’s second law as follows:

(34) $$\left\{\begin{array}{l} M{\ddot{x}}_{1}+K(2x_{1}-x_{3})+c(2{\dot{x}}_{1}-{\dot{x}}_{3})-2k(x_{2}-x_{1})-2k_{SMA}(x_{2}-x_{1})-2\varepsilon \Gamma {\left(x_{2}-x_{1}\right)}^{3}=F\sin(\omega t)\\ m{\ddot{x}}_{2}+2k(x_{2}-x_{1})+2k_{SMA}(x_{2}-x_{1})+2\varepsilon \Gamma {\left(x_{2}-x_{1}\right)}^{3}=0\\ M{\ddot{x}}_{3}+K(2x_{3}-x_{1}-x_{5})+c(2{\dot{x}}_{3}-{\dot{x}}_{1}-{\dot{x}}_{5})-2k(x_{4}-x_{3})-2k_{SMA}(x_{4}-x_{3})-2\varepsilon \Gamma {\left(x_{4}-x_{3}\right)}^{3}=0\\ m{\ddot{x}}_{4}+2k(x_{4}-x_{3})+2k_{SMA}(x_{4}-x_{3})+2\varepsilon \Gamma {\left(x_{4}-x_{3}\right)}^{3}=0\\ M{\ddot{x}}_{5}+K(2x_{5}-x_{3}-x_{7})+c(2{\dot{x}}_{5}-{\dot{x}}_{3}-{\dot{x}}_{7})-2k(x_{6}-x_{5})-2k_{SMA}(x_{6}-x_{5})-2\varepsilon \Gamma {\left(x_{6}-x_{5}\right)}^{3}=0\\ m{\ddot{x}}_{6}+2k(x_{6}-x_{5})+2k_{SMA}(x_{6}-x_{5})+2\varepsilon \Gamma {\left(x_{6}-x_{5}\right)}^{3}=0\\ M{\ddot{x}}_{7}+K(2x_{7}-x_{5}-x_{9})+c(2{\dot{x}}_{7}-{\dot{x}}_{5}-{\dot{x}}_{9})-2k(x_{8}-x_{7})-2k_{SMA}(x_{8}-x_{7})-2\varepsilon \Gamma {\left(x_{8}-x_{7}\right)}^{3}=0\\ m{\ddot{x}}_{8}+2k(x_{8}-x_{7})+2k_{SMA}(x_{8}-x_{7})+2\varepsilon \Gamma {\left(x_{8}-x_{7}\right)}^{3}=0\\ M{\ddot{x}}_{9}+K(x_{9}-x_{7})+c({\dot{x}}_{9}-{\dot{x}}_{7})-2k(x_{10}-x_{9})-2k_{SMA}(x_{10}-x_{9})-2\varepsilon \Gamma {\left(x_{10}-x_{9}\right)}^{3}=0\\ m{\ddot{x}}_{10}+2k(x_{10}-x_{9})+2k_{SMA}(x_{10}-x_{9})+2\varepsilon \Gamma {\left(x_{10}-x_{9}\right)}^{3}=0 \end{array}\right.$$

Here, $x_{1},x_{3},x_{5},x_{7,}x_{9}$ represent the displacement of the shell over one to five periods, while $x_{2},x_{4},x_{6},x_{8,}x_{10}$ represent the displacement of the inner Duffing oscillator over one to five periods.

The system of nonlinear differential equations given in Equation (34) is numerically solved in MATLAB using the ode45 solver with an adaptive time-stepping scheme. The relative and absolute error tolerances are set to $1\;\times {\;10}^{-5}$ and $1\;\times {\;10}^{-7}$, respectively. For each excitation frequency, the system is initially integrated from *t* = 0 to 1500 s with zero initial conditions to allow the transient response to decay sufficiently. The state at *t* = 1500 s is then used as the initial condition for a further integration over 1500 ≤ t ≤ 2000 s, from which the steady-state response is extracted.

During the steady-state interval, an event-detection procedure is employed to monitor the negative-going zero crossings of the velocities of the first and fifth outer shells. The corresponding displacement responses of the first and fifth shells are recorded, and their maximum values are taken as the input and output steady-state displacement amplitudes, denoted by $X_{in}$ and $X_{out}$, respectively. The vibration transmission is then calculated as

$$T_{dB}=20\log_{10}\left(\frac{X_{out}}{X_{in}}\right)$$

In the present state-variable formulation, $X_{in}$ and $X_{out}$ correspond to the displacements $x_{1}$ and $x_{9}$ of the first and fifth outer shells, respectively. The angular excitation frequency is swept according to *ω_j_* = 0.4*j*, *j* = 1, 2, … 190, corresponding to a frequency interval of approximately 0.0637 Hz and a frequency range of approximately 0.064–12.0 Hz. A harmonic excitation force Fsin(ωt), with *F* = 0.4 N, is applied to the outer shell of the first unit cell.

A fixed–free boundary condition is adopted for the five-period structure: the left end of the first unit cell is fixed, whereas the right end of the fifth unit cell is free. In the present reduced-order model, viscous damping with *c* = 0.05 Ns/m is introduced only in the coupling between adjacent outer shells, while the internal local resonators are assumed to be undamped. This simplified damping configuration is adopted to suppress transient responses while retaining the principal local-resonance characteristics. Additional material and interface damping in a practical structure may quantitatively affect the resonance amplitudes and attenuation depth.

It should also be noted that the very deep transmission minima and several narrow fluctuations inside the attenuation region may be sensitive to the finite integration duration, discrete frequency sampling, and the numerical extraction of extremely small steady-state responses. Therefore, these fine-scale features are not interpreted as independent physical phenomena in the present study; instead, the transmission curves are mainly used to determine the overall attenuation region and its temperature-dependent variation.

### 4.1. Effect of Temperature on the Starting and Cut-Off Frequencies of the Bandgap

**Figure 7:**
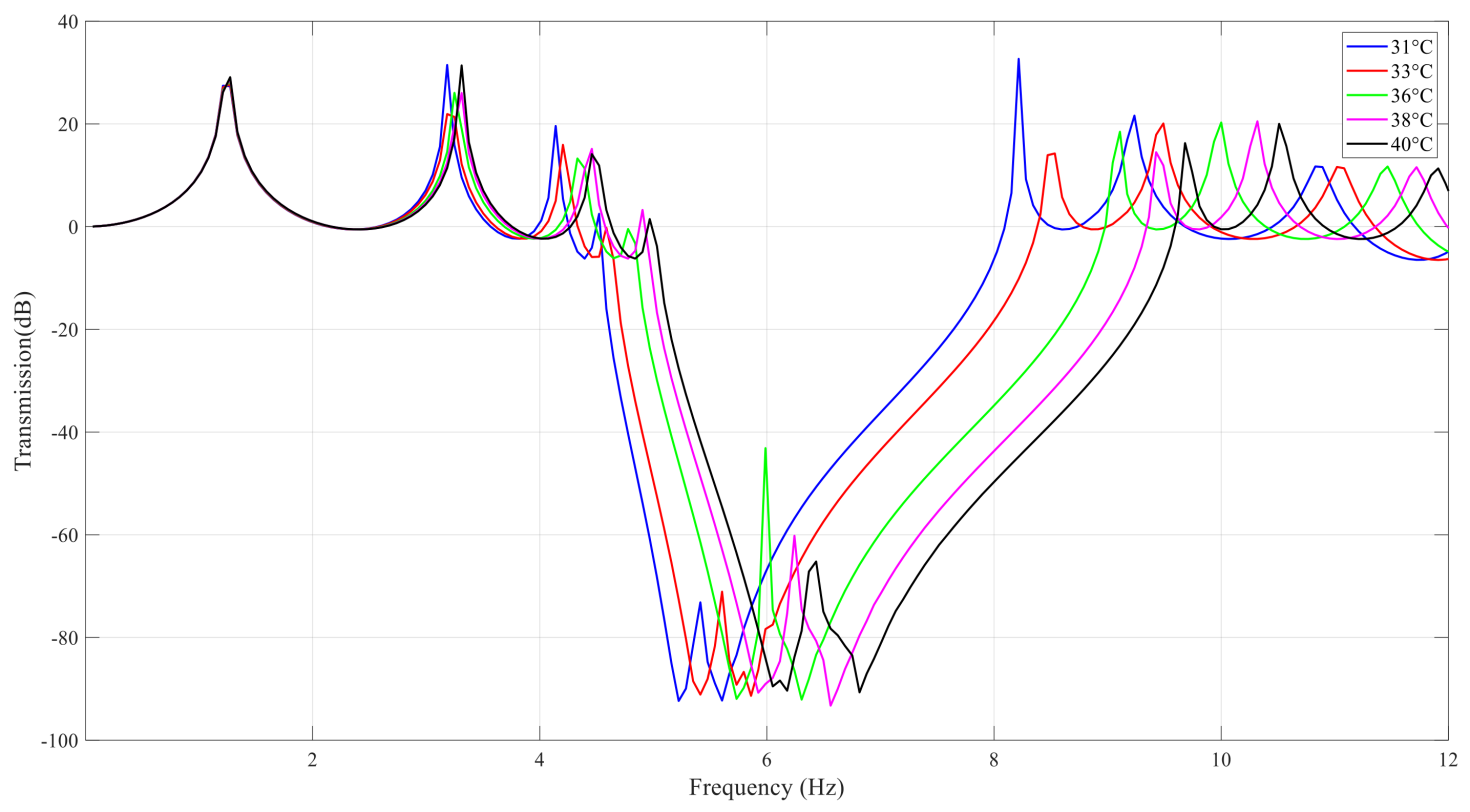
Effect of temperature variation on the bandgap range.

To verify the trends predicted by the theoretical analysis in the previous section regarding the effects of temperature variation on the starting frequency, cut-off frequency, and bandgap range, and to further demonstrate that the SMA spring can vary its stiffness through a temperature-induced phase transformation, thereby enabling active tuning of the bandgap range, the mass of the shell selected is *M* = 0.1 kg, the mass of the internal oscillator is *m* = 0.125 kg, the stiffness coefficient of the internal nonlinear spring is *k* = 37 N/m, the stiffness coefficient of the connecting spring between the shells is *K* = 117 N/m, the value of *F* is 0.4 N, and the damping coefficient of the external linear spring is *c* = 0.05 Ns/m. The nonlinear perturbation term is taken as *ε* = 0.01. With $M_{f}=29\;{}^{\circ}C$, $M_{s}=42\;{}^{\circ}C$, $A_{s}=43\;{}^{\circ}C$, $A_{f}=58\;{}^{\circ}C$ and the elastic modulus of full martensite being $E_{M}=35\;\mathrm{GPa}$, the elastic modulus of full austenite is $E_{A}=$ 70 GPa, and the Poisson’s ratio is ν=0.33 [29,30,38]. We set the wire diameter of the SMA helical spring to *d* = 0.001 m and the coil diameter to *D* = 0.01 m, with the number of active coils *N* taken as 50. The bandgap ranges are investigated at temperatures of 31 °C, 33 °C, 36 °C, 38 °C and 40 °C, respectively. The resulting vibration-transmission curves are shown in Figure 7:

Figure 7 presents the vibration-transmission responses at different temperatures. The corresponding starting frequencies, cut-off frequencies, and bandwidths are summarized in Table 1. During cooling, both the starting and cut-off frequencies shift toward lower frequencies, accompanied by a decrease in bandgap width.

The above variations are primarily attributed to the temperature-dependent stiffness of the SMA spring. The present study focuses on the cooling process. As the temperature decreases, the SMA undergoes a phase transformation, accompanied by a variation in its elastic modulus, which consequently leads to a change in the stiffness *k_SMA_* of the SMA spring. Since the SMA spring and the internal Duffing oscillator jointly constitute the local-resonance unit, the variation in the SMA spring stiffness directly alters the effective connecting stiffness of the local resonator and further affects the local-resonance frequency of the system. Consequently, these changes are manifested in the vibration-transmission characteristics as shifts in the attenuation region and the corresponding bandgap position.

When *T* = 38 °C, the resonance frequency of the internal oscillator is:

(35) $$f_{r}=\frac{1}{2\pi }\sqrt{\frac{2(k+k_{SMA})}{m}}=\frac{1}{2\pi }\sqrt{\frac{2\times (37+58.7)}{0.125}}\approx 6.23\;\mathrm{Hz}$$

According to Equation (35), the local-resonance frequency of the internal Duffing oscillator is calculated to be approximately 6.23 Hz. This frequency represents an important characteristic frequency at which the dynamic response of the local-resonance unit undergoes a pronounced change, and it also serves as a key reference for analyzing the different effective-mass characteristics within the bandgap.

### 4.2. Temperature-Dependent Bandgap-Tuning Behavior

As summarized in Table 1, cooling from 40 °C to 31 °C decreases the starting frequency from 4.98 Hz to 4.53 Hz and the cut-off frequency from 9.60 Hz to 8.09 Hz, corresponding to reductions of approximately 9.04% and 15.73%, respectively. The bandwidth decreases from 4.62 Hz to 3.56 Hz, representing a reduction of approximately 22.94%. These results demonstrate that cooling shifts the attenuation frequency range toward lower frequencies while narrowing its bandwidth, allowing the attenuation range to be adjusted through temperature control.

Previously reported Duffing-based acoustic metamaterials exhibit band-gap characteristics that depend on structural parameters and excitation amplitude [22]. Under fixed structural parameters and excitation conditions, however, these models do not provide a temperature-controlled stiffness-tuning mechanism. The present metamaterial introduces this additional tuning capability through the SMA springs. Changing the temperature modifies the SMA stiffness and shifts the band-gap frequency range without altering the geometry, mass parameters, or excitation conditions. The temperature-dependent transmission responses in Figure 7 and the results summarized in Table 1 illustrate the corresponding shift in the finite-chain attenuation range.

### 4.3. Responses of the Shells and Internal Oscillators in the Bandgap Region

For ease of comparison of the dynamic responses of the system under different excitation frequencies, *T* = 38 °C is fixed in the subsequent analysis, and representative frequencies within the corresponding bandgap and passband ranges at this temperature are selected for investigation.

**Figure 8:**
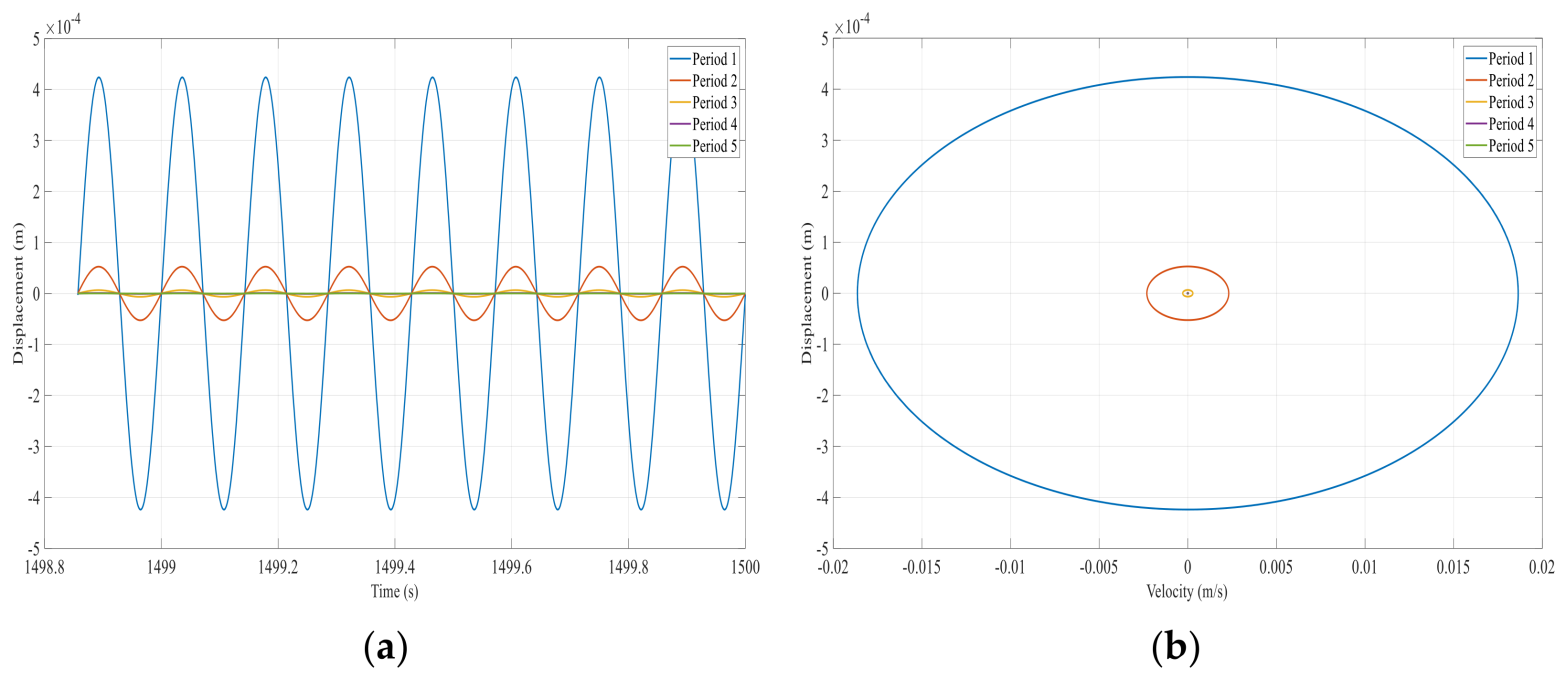
Five-period shells: Displacement–time history curves (**a**) and displacement–velocity phase diagrams (**b**). (*f* = 7 Hz).

**Figure 9:**
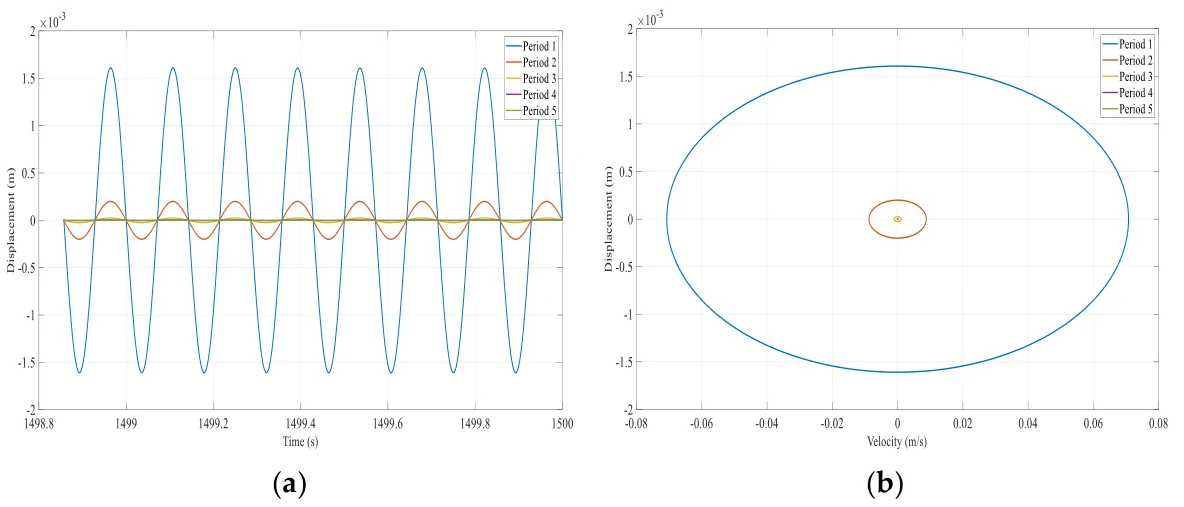
Five-period internal oscillators: Displacement–time history curves (**a**) and displacement–velocity phase diagrams (**b**). (*f* = 7 Hz).

To verify the suppression of vibration propagation within the bandgap and to analyze the effective dynamic characteristics of the system above the local-resonance frequency, an external excitation frequency of *f* = 7 Hz is selected for numerical analysis. The vibration displacement–time history curves and phase diagrams of the shells and internal oscillators in the five period cells are plotted using MATLAB, as shown in Figure 8 and Figure 9:

At *f* = 7 Hz, the responses of both the shells and internal oscillators gradually approach stable periodic motion after the initial transient process, as shown in Figure 8 and Figure 9. The corresponding displacement–velocity trajectories form closed and relatively regular curves, indicating that the five-period structure has reached a steady state. More importantly, the displacement amplitudes decrease progressively from the first period cell to the fifth for both the shells and internal oscillators. This spatial attenuation confirms that vibration propagation is strongly suppressed at this excitation frequency, which is consistent with its location inside the bandgap.

Regarding the vibration transmission across the five period cells, we analyze the steady-state displacement amplitudes of the shells and internal oscillators. As illustrated in Figure 8a, the displacement amplitude of the first period cell is approximately $4.2\;\times \;10^{-4}$ m, whereas that of the second period cell is only about $5.2\;\times \;10^{-5}$ m. It further attenuates to approximately $6.5\;\times {\;10}^{-6}$ m in the third period cell, $8\;\times \;10^{-7}$ m in the fourth period cell, and only about $1.1\;\times \;10^{-7}$ m in the fifth period cell.

Similarly, the vibration behavior of the internal Duffing oscillators closely resembles that of the shells, with the displacement amplitudes across the period cells also exhibiting a trend of progressive attenuation. As shown in Figure 9a, the displacement amplitude in the first period cell is approximately $1.6\;\times {\;10}^{-3}$ m, whereas that in the second period cell is only about $1.9\;\times \;10^{-4}$ m. It attenuates to approximately $2.47\;\times \;10^{-5}$ m in the third period cell, $3\times 10^{-6}$ m in the fourth period cell, and only about $4.2\;\times \;10^{-7}$ m in the fifth period cell. From the displacement amplitudes of the shells and internal oscillators, it is evident that the vibration displacement continuously attenuates. This phenomenon is consistent with the characteristics of a bandgap.

**Figure 10:**
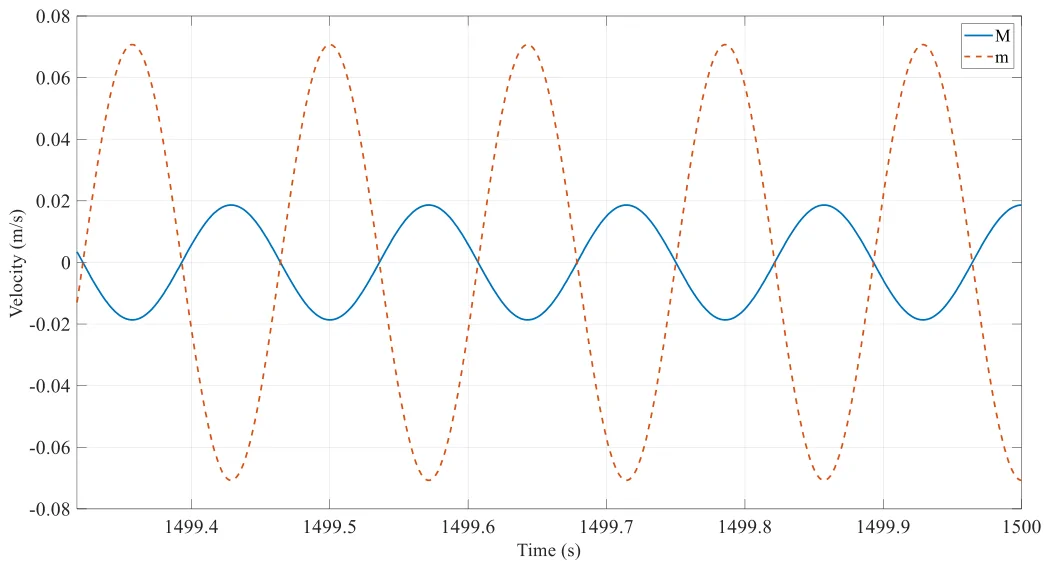
First period shell and internal oscillator: Velocity–time history curves. (*f* = 7 Hz).

Next, the velocity–time history curves of the shell and the internal oscillator in the first period cell after reaching steady-state motion are plotted, as shown in Figure 10:

The dynamic origin of the attenuation at *f* = 7 Hz can be further identified from the velocity responses in Figure 10. The shell and the internal Duffing oscillator move predominantly in opposite directions, showing a clear out-of-phase relationship. Together with the adopted mass parameters and the larger velocity amplitude of the internal oscillator, this motion leads to a total momentum opposite to the velocity of the shell, corresponding to an ENM state.

To further clarify the physical mechanism of the vibration attenuation shown in Figure 10, the effective mass of the locally resonant unit is introduced. For the mass-in-mass system considered in this study, the effective mass can be expressed as $M_{\mathrm{eff}}(\omega )=M+\frac{mf_{r}^{2}}{f_{r}^{2}-\omega^{2}}$,where *M* and *m* denote the masses of the outer shell and the internal oscillator, respectively, and *ω*_0_ is the local-resonance frequency determined by the equivalent stiffness of the internal resonator [39].

For the parameters considered in Figure 10, *M* = 0.1 kg, *m* = 0.125 kg, and the local-resonance frequency is $f_{r}=6.23\;\mathrm{HZ}$. At the excitation frequency *f* = 7 Hz, therefore, $M_{\mathrm{eff}}<0$, indicating that the system is in the ENM region at 7 Hz.

This quantitative result is consistent with the dynamic responses shown in Figure 10. The velocity responses of the outer shell and the internal oscillator exhibit an approximately opposite phase relationship, indicating that the internal oscillator moves against the motion of the outer shell near the local resonance. Such relative motion produces an inertial effect opposite to the external excitation and is characteristic of the ENM state.

Therefore, in the revised manuscript, the effective-mass state is determined quantitatively according to the analytical expression of *M*_eff_, while the velocity responses shown in Figure 10 are used to illustrate the corresponding dynamic behavior rather than used as the sole criterion for identifying the sign of the effective mass.

**Figure 11:**
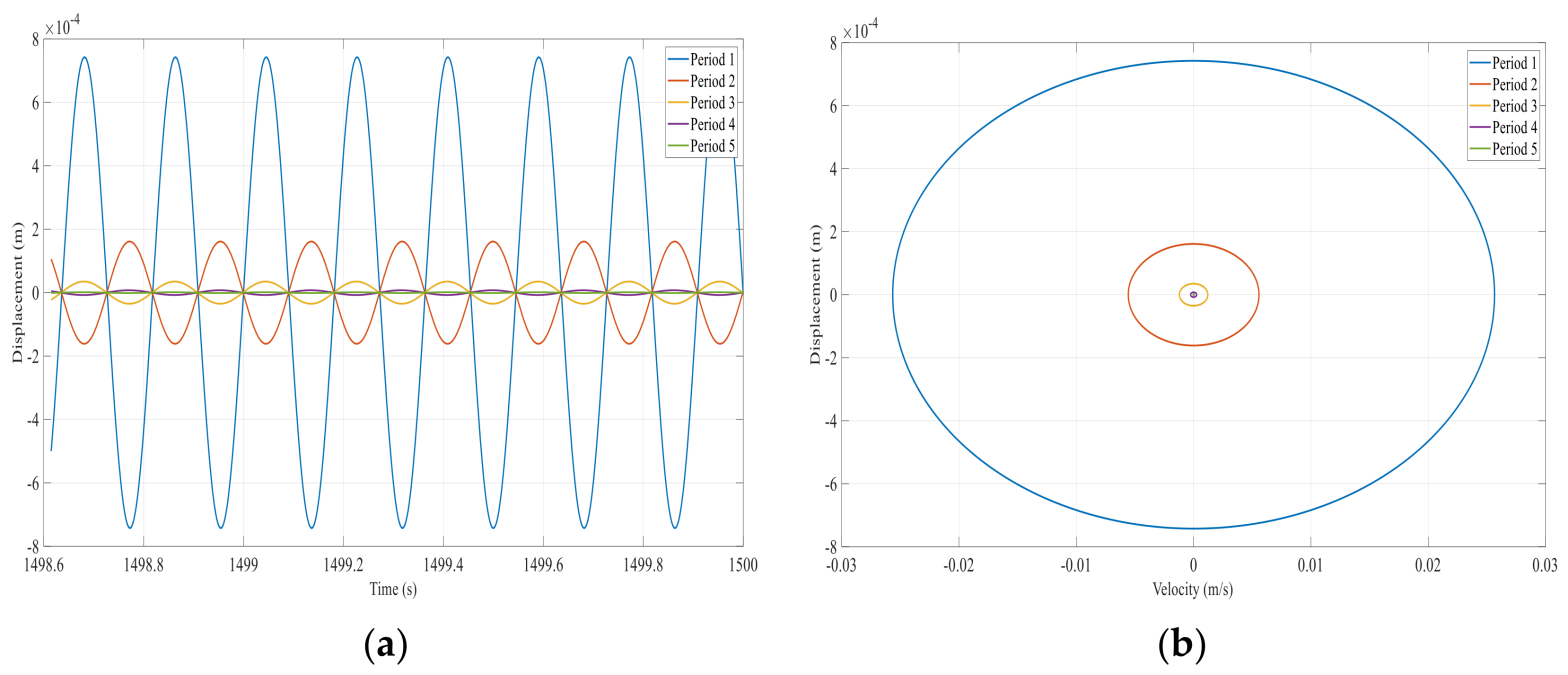
Five-period shells: Displacement–time history curves (**a**) and displacement–velocity phase diagrams (**b**). (*f* = 5.5 Hz).

**Figure 12:**
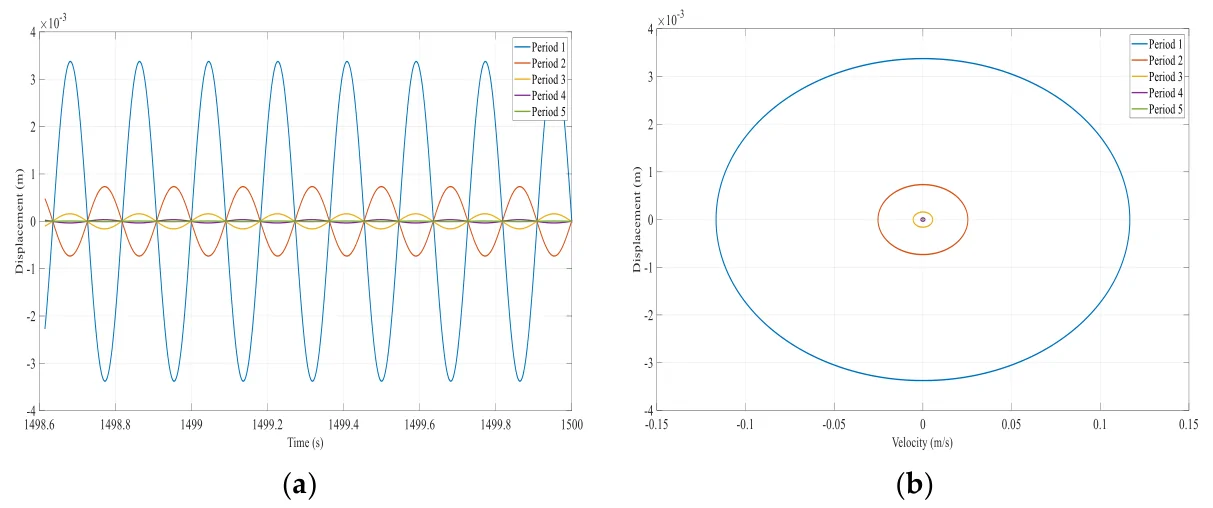
Five-period internal oscillators: Displacement–time history curves (**a**) and displacement–velocity phase diagrams (**b**). (*f* = 5.5 Hz).

With the external excitation frequency set to *f* = 5.5 Hz, the vibration displacement–time history curves and phase diagrams of the shells and internal oscillators in the five period cells are plotted, as shown in Figure 11 and Figure 12:

Figure 11 presents the displacement–time history curves and displacement–velocity phase diagrams of the shells in the five period cells of the SMA metamaterial model, while Figure 12 presents the displacement–time history curves and displacement–velocity phase diagrams of the internal oscillators in the five period cells. An analysis of the displacement–time history curves demonstrates an initial transient phase, after which the vibration amplitudes for both the shells and internal oscillators stabilize at constant levels. Furthermore, the corresponding displacement–velocity phase diagrams trace out relatively regular ellipses. This dynamic behavior confirms that all structural components have successfully transitioned into steady-state motion, completely avoiding any irregular or chaotic states.

For the vibration transmission across the five period cells, the steady-state displacement amplitudes of both the shells and internal oscillators exhibit progressive attenuation. As shown in Figure 11a and Figure 12a, the vibration amplitudes decrease continuously from the first period cell to the fifth period cell. The attenuation observed in both components indicates that vibration propagation is suppressed along the periodic structure, which is consistent with the characteristics of a bandgap.

**Figure 13:**
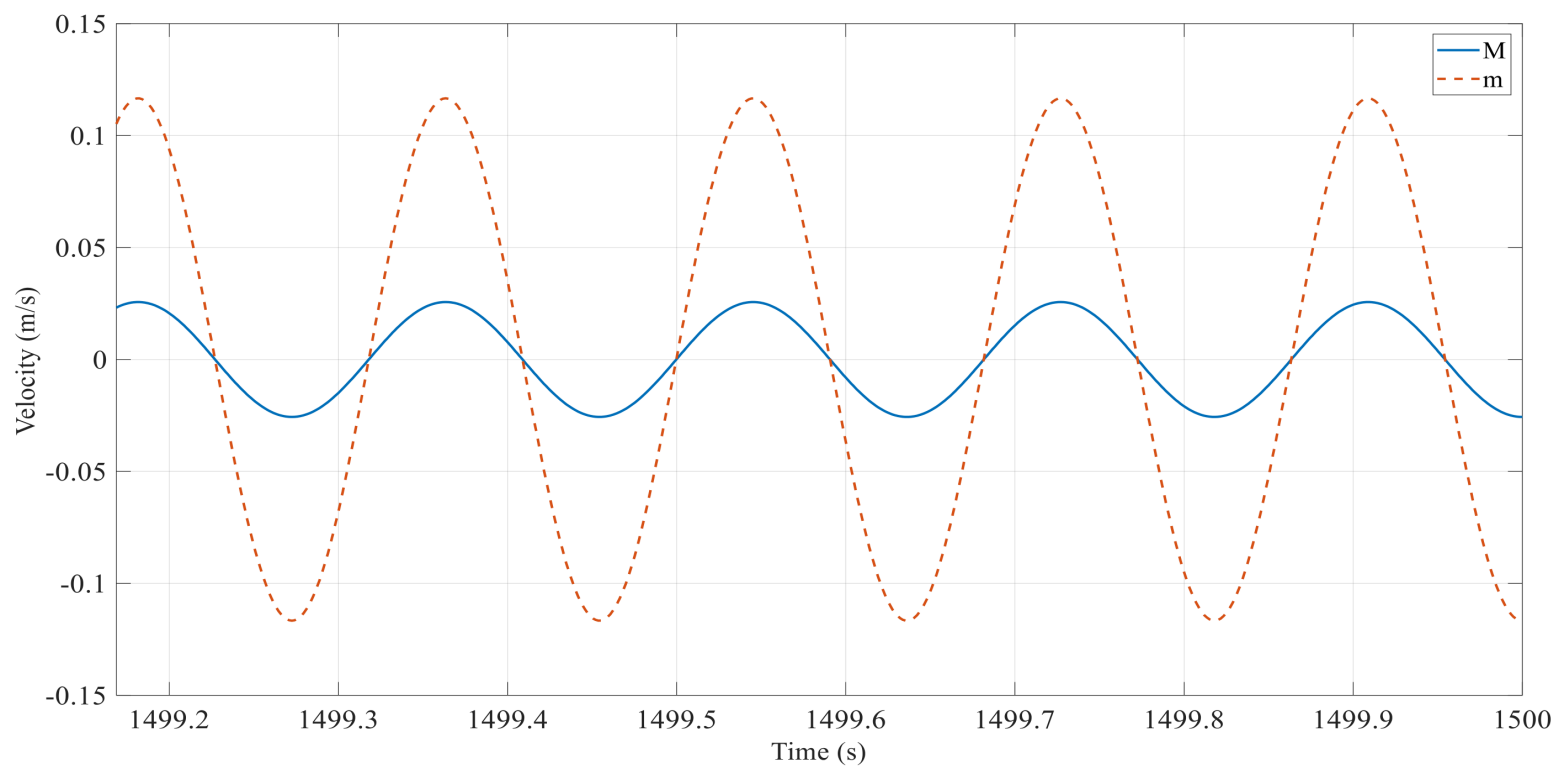
First period shell and internal oscillator: velocity–time history curves. (*f* = 5.5 Hz).

The effective-mass characteristic at *f* = 5.5 Hz is further examined using the velocity responses of the first period cell. The corresponding velocity–time histories of the shell and internal oscillator are shown in Figure 13:

Figure 13 presents the steady-state velocity–time history curves of the shell and the internal oscillator in the first period cell at an excitation frequency of *f* = 5.5 Hz. When the velocity of the shell *M* reaches its positive peak, the velocity of the internal oscillator *m* also reaches its positive peak. In other words, the velocity responses of the shell and the internal oscillator exhibit a pronounced in-phase relationship. Further analysis shows that the velocities of the shell and the internal oscillator are essentially in the same direction; hence, their momenta are also directed in the same direction, resulting in a positive total momentum of the period cell. Since the velocity of the shell is taken as positive, the relationship between the total momentum and the shell velocity indicates that the system exhibits an effective-positive-mass characteristic. Nevertheless, the system remains within a frequency range in which vibration propagation is significantly suppressed. This demonstrates that the bandgap of the acoustic metamaterial investigated in this study does not consist solely of an ENM region, but also contains a dynamic response region characterized by effective positive mass.

### 4.4. Responses of the Shells and Internal Oscillators Within the Passband

**Figure 14:**
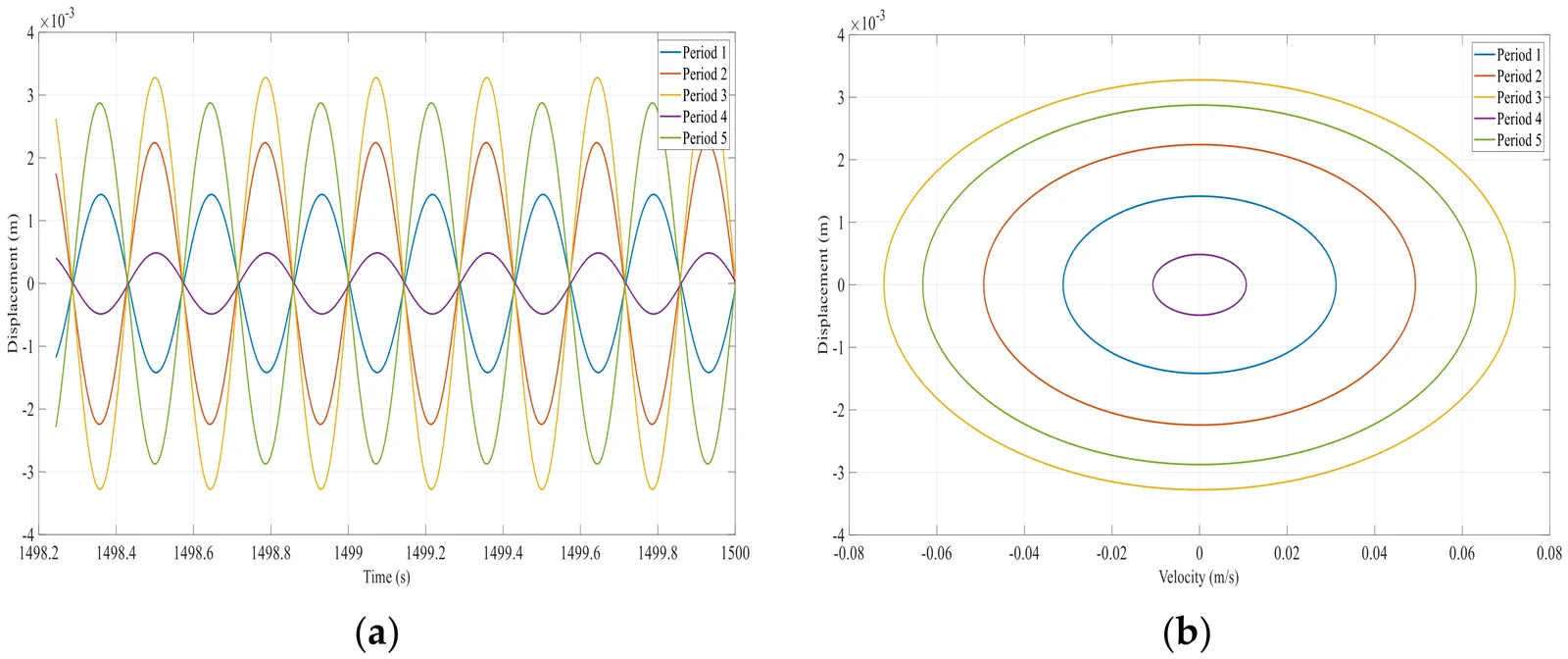
Five-period shells: Displacement–time history curves (**a**) and displacement–velocity phase diagrams (**b**). (*f* = 3.5 Hz).

**Figure 15:**
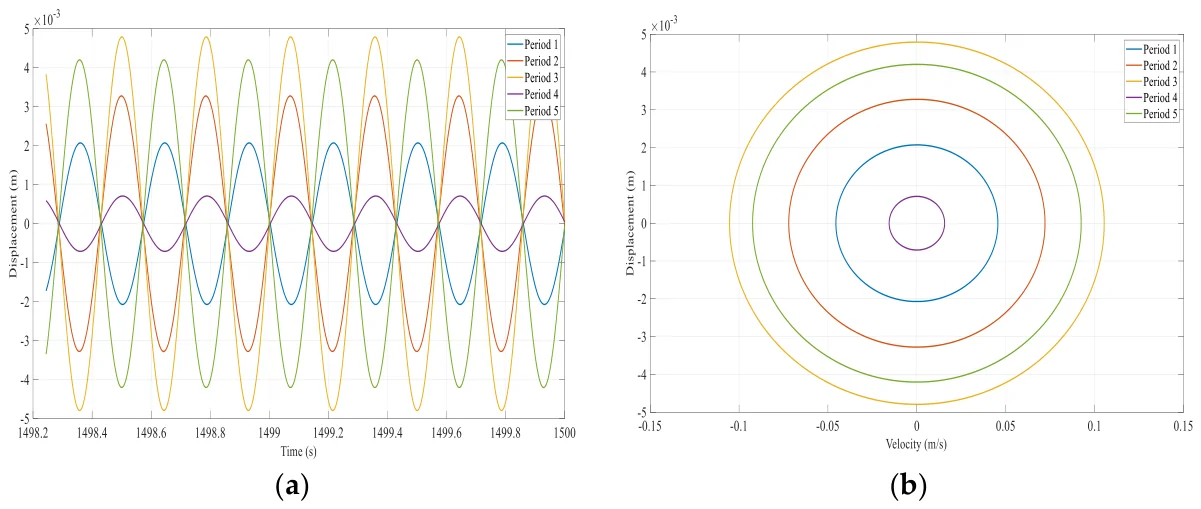
Five-period internal oscillators: Displacement–time history curves (**a**) and displacement–velocity phase diagrams (**b**). (*f* = 3.5 Hz).

The preceding analysis demonstrates that the acoustic metamaterial model incorporating SMAs exhibits both effective-positive-mass and ENM characteristics within the bandgap range, which is consistent with the aforementioned theoretical analysis. Next, still setting the temperature as *T* = 38 °C and the external excitation frequency as 3.5 Hz, the displacement–time history curves and displacement–velocity phase diagrams for the shells and internal oscillators at this frequency are plotted, as shown in Figure 14 and Figure 15:

Figure 14 and Figure 15 show that, after the transient response, both the shells and internal oscillators reach stable periodic motion. Their displacement–velocity phase trajectories form relatively regular closed curves, indicating that no irregular or chaotic response occurs. For the shells, the displacement amplitudes do not decrease progressively from the first to the fifth period cell; instead, local increases in amplitude can be observed in the subsequent cells. This behavior indicates that vibration is transmitted through the periodic structure rather than being continuously attenuated.

A similar trend is observed for the internal Duffing oscillators in Figure 15a. Their displacement amplitudes also do not exhibit progressive attenuation along the five period cells, showing that the excitation can propagate from the first period cell to the downstream cells. The responses of both the shells and internal oscillators therefore exhibit the typical transmission characteristics of a passband.

**Figure 16:**
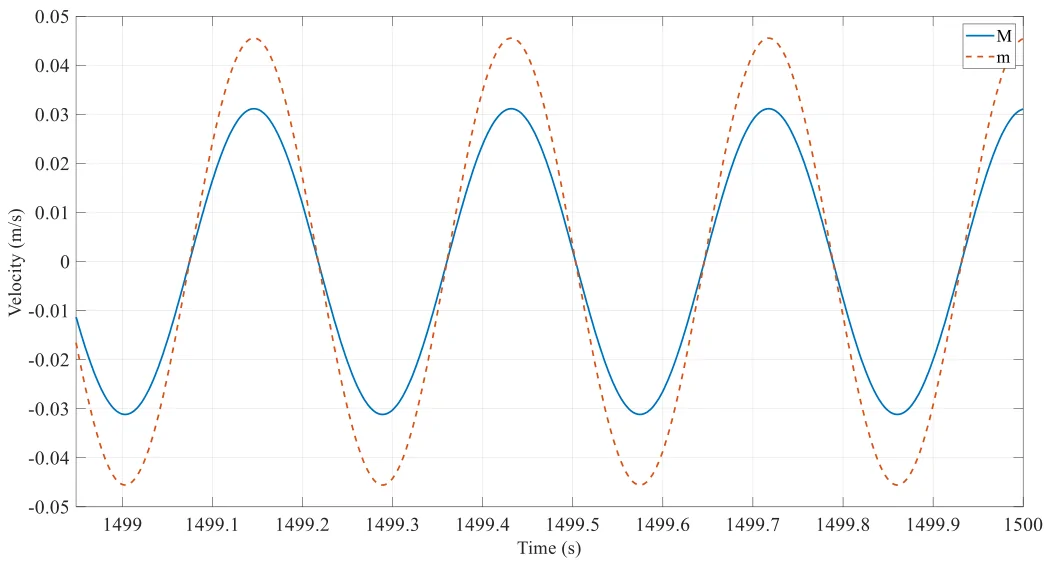
First period shell and internal oscillator: Velocity–time history curves. (*f* =3.5 Hz).

Next, the velocity–time history curves of the shell and the internal oscillator in the first period cell at *f* = 3.5 Hz are plotted, as shown in Figure 16:

Figure 16 compares the velocity–time histories of the shell *M* and the internal Duffing oscillator *m* in the first period cell at *f* = 3.5 Hz. When the velocity of the shell reaches a positive peak, the internal oscillator also reaches a positive peak; similarly, their negative peaks occur in the same direction. Thus, the shell and the internal oscillator exhibit an in-phase motion. Accordingly, the acoustic metamaterial with SMA springs exhibits an effective-positive-mass characteristic at this frequency.

### 4.5. Zero-Mass Phenomenon at the Bandgap Cut-Off Frequency

**Figure 17:**
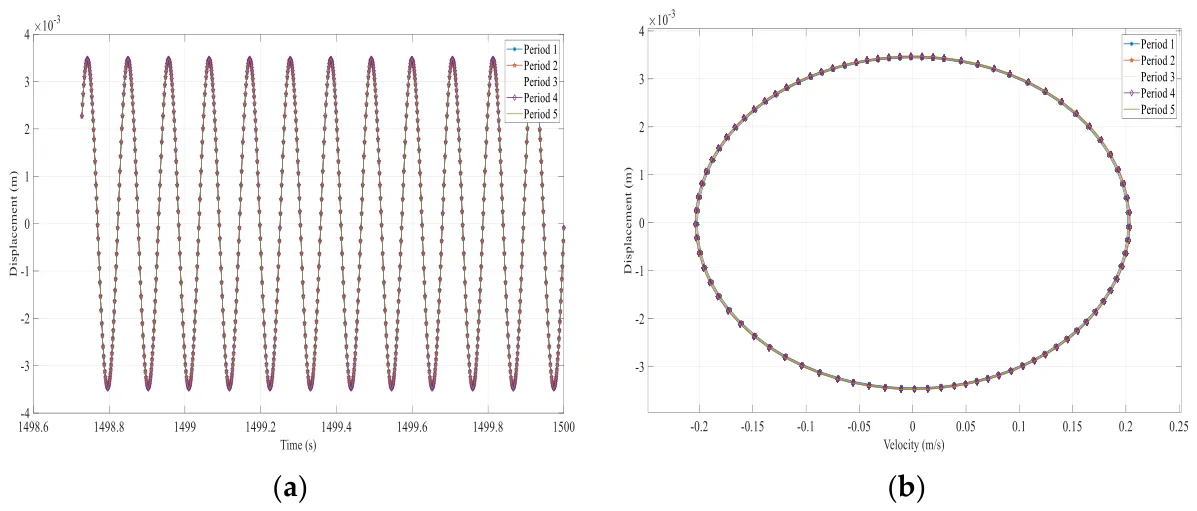
Zero-mass five-period shells: Displacement–time history curves (**a**) and displacement–velocity phase diagrams (**b**). (*f* = 9.342 Hz).

**Figure 18:**
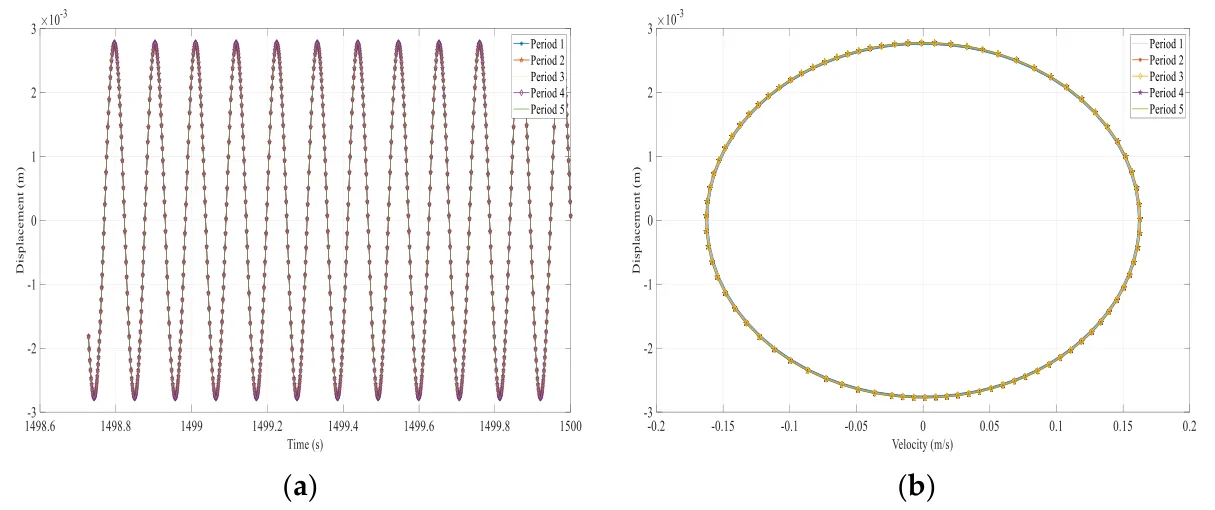
Zero-mass five-period internal oscillators: Displacement–time history curves (**a**) and displacement–velocity phase diagrams (**b**). (*f* = 9.342 Hz).

Building on the preceding analysis of the dynamic characteristics associated with effective positive and negative mass within the bandgap, this section further investigates the zero-effective-mass state of the system at the bandgap boundary. For the local-resonance periodic structure, the frequency corresponding to zero-effective-mass coincides with the cut-off frequency of the bandgap, indicating a direct correspondence between the zero-effective-mass frequency and the bandgap cut-off frequency. Since the period cell considered in this study contains a Duffing-type nonlinear spring, the dynamic characteristics of the system exhibit a certain degree of amplitude dependence. Consequently, the nonlinear effect may cause the characteristic frequencies observed in the actual dynamic response to deviate slightly from those predicted by the linear theory [22]. Therefore, a refined calculation with dense frequency sampling is performed in the vicinity of the bandgap cut-off frequency to accurately determine the cut-off frequency and, correspondingly, the zero-effective-mass frequency. The zero-effective-mass frequency is determined to be 9.342 Hz, and the motion characteristics of each period cell at this frequency are further analyzed. The corresponding displacement–time history curves and displacement–velocity phase diagrams of the shells and internal Duffing oscillators in the five-period structure are shown in Figure 17 and Figure 18:

Figure 17 and Figure 18 show that the shells and internal oscillators in all period cells are in continuous motion. Furthermore, the phase diagrams of the shells and internal oscillators do not exhibit any irregular or chaotic motion states, indicating that they have achieved steady-state motion, and their maximum displacements are nearly identical.

**Figure 19:**
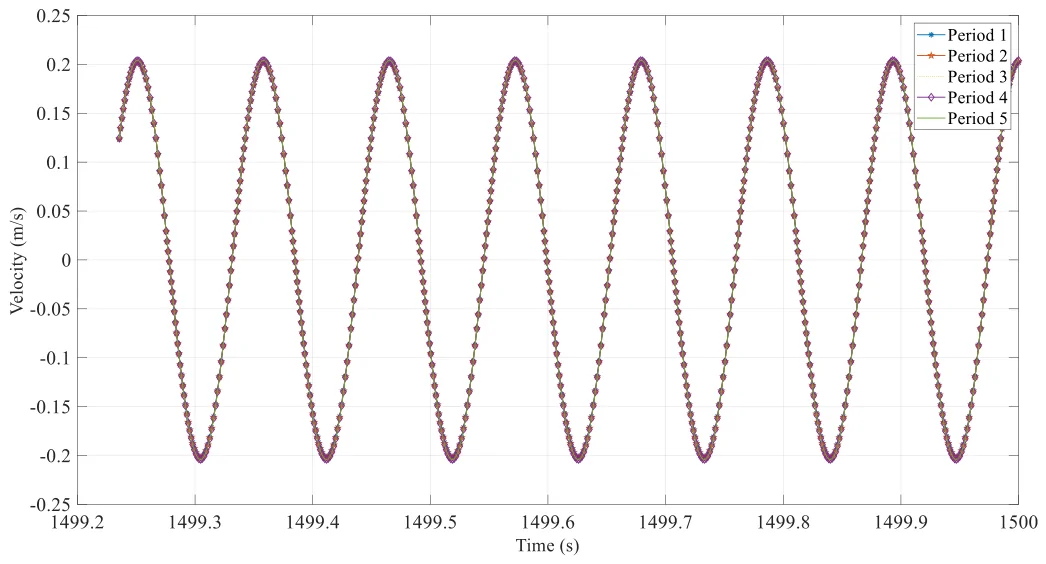
Zero-mass five-period shells: Velocity–time history curves. (*f* = 9.342 Hz).

**Figure 20:**
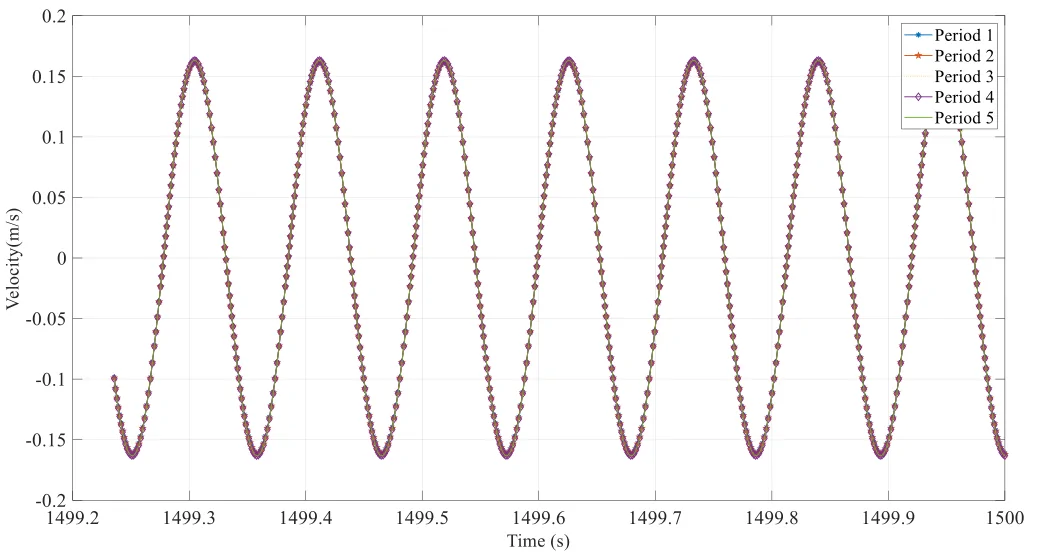
Zero-mass five-period internal oscillators: Velocity–time history curves. (*f* = 9.342 Hz).

To further examine the motion characteristics under the zero-effective-mass condition, the velocity–time histories of the shells and internal oscillators in the five period cells are compared in Figure 19 and Figure 20, respectively.

As can be observed from Figure 19, the velocity–time history curves of the shells in the five period cells almost completely overlap, indicating that the shells exhibit nearly equal amplitude and in-phase motion. Consequently, the connecting springs *K* between adjacent period cells undergo negligible relative deformation. Figure 20 also shows that the internal Duffing oscillators in different period cells exhibit nearly identical in-phase responses. However, within each individual period cell, the internal oscillator moves out of phase with the corresponding shell. Therefore, the zero-effective-mass state should not be interpreted as a pure translational motion of the entire system. For the fixed–free finite chain considered here, the zero-effective-mass condition corresponds to equal shell amplitudes, satisfying *X* = *F/K*, while the relative motion between the shell and the internal oscillator remains present.

The determination of the zero-effective-mass frequency provides a clear characteristic parameter for bandgap tuning in acoustic metamaterials incorporating SMA springs. Since the stiffness of the SMA spring varies with temperature, changes in temperature will shift both the zero-effective-mass frequency and the corresponding bandgap cut-off frequency. Therefore, by adjusting the temperature of the SMA spring, active tuning of the bandgap cut-off frequency and the effective vibration isolation frequency range can be achieved.

## 5. Conclusions

This study develops a temperature-tunable local-resonance acoustic metamaterial incorporating SMA springs for adaptive vibration attenuation. By introducing SMA springs into the internal structure, the effective connecting stiffness of the local-resonance unit can be continuously varied with ambient temperature, thereby enabling active tuning of the dispersion characteristics and bandgap range. Based on the perturbation method, the first-order perturbation solutions for the acoustic and optical branches under weakly nonlinear conditions are derived. Combined with the vibration-transmission characteristics of a finite-period structure, the bandgap position, bandgap width, and the effective-positive-mass, ENM, and zero-effective-mass characteristics within the bandgap at different temperatures are systematically analyzed. The results demonstrate that the SMA springs can convert temperature variations into changes in the internal stiffness parameters of the periodic structure, thereby enabling active tuning of the bandgap frequency range without altering the structural geometry or mass parameters. Finally, the proposed SMA-based nonlinear acoustic metamaterial provides a feasible strategy for developing adaptive functional materials with temperature-controlled vibration attenuation capability, which may offer potential applications in low-frequency vibration isolation, structural noise reduction, and intelligent vibration control systems. The main conclusions are summarized as follows:

(1) For the periodic acoustic metamaterial incorporating SMA springs, the dimensionless dispersion relation is dependent on the stiffness ratio between the SMA spring and the nonlinear spring. As the temperature decreases, both the acoustic and optical branches vary and shift toward the lower-frequency range.
(2) For the ENM acoustic metamaterial incorporating SMA springs, the starting frequency, cut-off frequency, and width of the bandgap can be actively tuned by adjusting the temperature of the SMA springs. As the temperature decreases, the bandgap shifts toward the lower-frequency range and simultaneously becomes narrower.
(3) Consistent with the established effective-mass theory of classical mass-in-mass locally resonant lattices [21,22], the bandgap of the present structure contains an effective-positive-mass attenuation region below the local-resonance frequency and an ENM region above the local-resonance frequency. In the proposed SMA-based system, temperature-dependent variation in the SMA spring stiffness modifies the local-resonance frequency and consequently shifts the frequency ranges associated with these established effective-mass regimes. The finite-period simulations further illustrate the corresponding temperature-dependent vibration attenuation behavior.
(4) In the present SMA-based metamaterial, the temperature-dependent variation in the SMA spring stiffness changes the local-resonance frequency and consequently shifts both the zero-effective-mass frequency and the corresponding bandgap cut-off frequency.
(5) The incorporation of SMA springs provides an additional temperature-controlled mechanism for band-gap tuning. With the geometry, mass parameters, and excitation conditions unchanged, varying the temperature shifts the band-gap frequency range through changes in the SMA stiffness. This capability extends the tuning options of fixed-stiffness Duffing metamaterials and offers a means of adjusting the vibration attenuation range without structural redesign.

## Figures and Tables

**Table 1:** Temperature-dependent attenuation frequency ranges of the SMA-based nonlinear metamaterial.

Temperature (°C)	Starting Frequency (Hz)	Cut-Off Frequency (Hz)	Bandgap Width (Hz)
40	4.98	9.60	4.62
38	4.92	9.34	4.42
36	4.77	8.97	4.20
33	4.58	8.38	3.80
31	4.53	8.09	3.56

## Data Availability

The original contributions presented in this study are included in the article material. Further inquiries can be directed to the corresponding authors.
